# Motor network organization in healthy development and chronic tic disorders

**DOI:** 10.1093/braincomms/fcaf260

**Published:** 2025-06-30

**Authors:** Theresa V Heinen, Julia Schmidgen, Theresa Paul, Lukas Hensel, Gereon R Fink, Lukas J Volz, Christian Grefkes, Stephan Bender, Kerstin Konrad

**Affiliations:** Child Neuropsychology Section, Department of Child and Adolescent Psychiatry, Psychosomatics and Psychotherapy, Medical Faculty, RWTH Aachen University, Aachen 52074, Germany; JARA-BRAIN Institute II, Molecular Neuroscience and Neuroimaging (INM-11), Forschungszentrum Jülich GmbH and RWTH Aachen University, Juelich 52428, Germany; Department of Child and Adolescent Psychiatry, Psychosomatics and Psychotherapy, Faculty of Medicine and University Hospital Cologne, University of Cologne, Cologne 50931, Germany; Medical Faculty, University of Cologne, and Department of Neurology, University Hospital Cologne, Cologne 50937, Germany; Medical Faculty, University of Cologne, and Department of Neurology, University Hospital Cologne, Cologne 50937, Germany; Medical Faculty, University of Cologne, and Department of Neurology, University Hospital Cologne, Cologne 50937, Germany; Institute of Neuroscience and Medicine, Cognitive Neuroscience (INM-3), Research Centre Juelich, Juelich 52425, Germany; Medical Faculty, University of Cologne, and Department of Neurology, University Hospital Cologne, Cologne 50937, Germany; Department of Neurology, Goethe University Frankfurt and University Hospital, Frankfurt 60528, Germany; Department of Child and Adolescent Psychiatry, Psychosomatics and Psychotherapy, Faculty of Medicine and University Hospital Cologne, University of Cologne, Cologne 50931, Germany; Child Neuropsychology Section, Department of Child and Adolescent Psychiatry, Psychosomatics and Psychotherapy, Medical Faculty, RWTH Aachen University, Aachen 52074, Germany; JARA-BRAIN Institute II, Molecular Neuroscience and Neuroimaging (INM-11), Forschungszentrum Jülich GmbH and RWTH Aachen University, Juelich 52428, Germany

**Keywords:** chronic motor tics, brain motor network organization, dynamic causal modelling (DCM), typical and atypical development

## Abstract

Tic disorders (TD) are childhood-onset neurodevelopmental disorders characterized by sudden, repetitive motor and vocal tics, often with partial or complete remission by the time young adulthood is reached. We here investigated motor control and compensatory neural processes in drug-naïve children and adolescents with chronic Motor Tic Disorder or Tourette Syndrome (TD) by examining motor network activity and connectivity compared to healthy controls. Using a reaction time (RT) task under varying cueing conditions, combined with functional magnetic resonance imaging (fMRI) and dynamic causal modelling (DCM), we explored how TD-related motor networks adapt to support volitional movement control. Participants with TD demonstrated enhanced task accuracy across internally and externally cued conditions despite deficits in sustained motor inhibition (blink suppression). Relative to controls, individuals with TD exhibited increased task-related activation in ipsilateral motor regions, particularly in the primary motor cortex, and somatosensory cortex, and enhanced interhemispheric connectivity between parietal sensory-motor hubs. Notably, while in typically developing participants, age-related increases in parietal lobe activation and modulatory connectivity between primary motor and premotor regions were linked to improved task accuracy, working memory and visuomotor coordination, TD patients deviated from this normative developmental trajectory with distinct, atypical but neither delayed nor accelerated neural activation and connectivity patterns. Our data suggest that TD involves compensatory neuroplastic adaptations that leverage additional sensorimotor resources to improve motor control but do not extend to motor inhibition processes. Moreover, the findings emphasize the intricate interplay between motor control and neural plasticity in TD, highlighting how compensatory mechanisms may serve as adaptive responses to motor challenges. These findings open avenues for therapeutic strategies that harness the brain's compensatory capacities to enhance motor control and facilitate TD management.

## Teaser

Heinen *et al*. use functional MRI and dynamic causal modelling to study motor control in typically developing children and adolescents and drug-naïve patients with Tic Disorders. Patients exhibit distinct brain activation and connectivity patterns, reflecting compensatory adaptations that support reactive motor control but diverge from normative developmental trajectories.

## Graphical Abstract

**Graphical Abstract:**
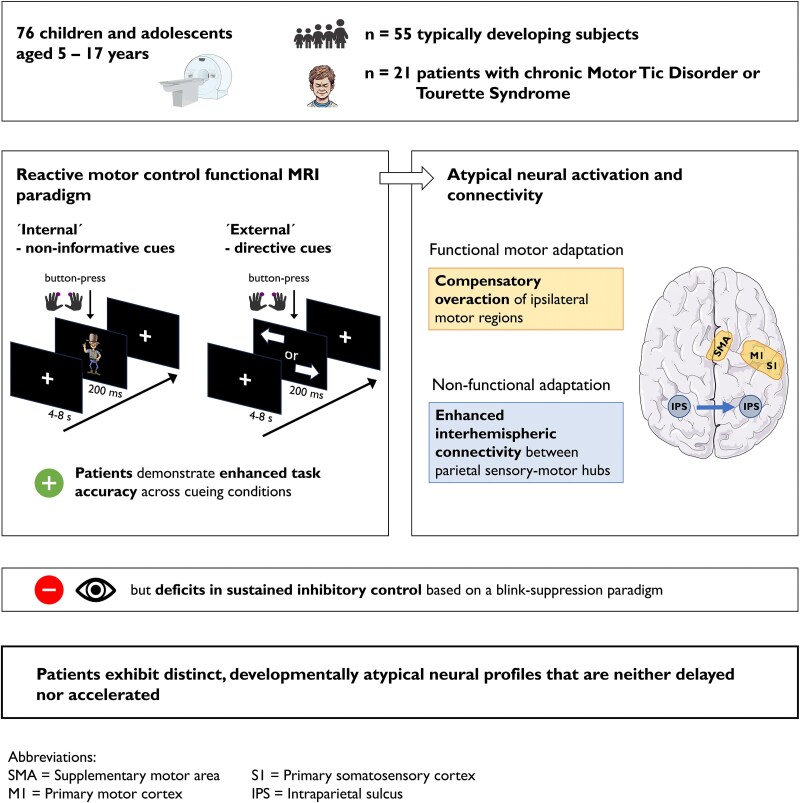

## Introduction

Tic disorders (TD) are childhood-onset movement disorders characterized by sudden, recurrent movements or vocalisations. These tics typically emerge during childhood, peaking between 9 and 11 years, with many children experiencing partial or complete remission as they approach early adulthood.^1-3^ TD are considered to encompass a spectrum of interconnected conditions, including Tourette Syndrome (TS) and chronic Motor Tic Disorder, with TS generally regarded as the more severe manifestation. However, both are recognized as expressions of a unified disease entity.^4,5^ While spontaneous remission of TD is common, the underlying mechanisms remain poorly understood.^2,6^

Recent research highlights the cortico-striatal-thalamo-cortical circuitry as a primary contributor to tic generation, proposing that disorganized network connectivity may result in disinhibition, leading to motor cortex hyperexcitability.^7-13^ Two competing hypotheses have been put forward to explain tic discontinuation: (i) development of neuroplastic compensatory mechanisms in frontal and motor networks that adaptively enhance motor control over time^14-16^ and (ii) delayed neurodevelopmental normalization of cortico-striatal-thalamo-cortical circuits.^17,18^ Notably, these hypotheses imply distinct pathways and developmental trajectories for motor network organization underlying motor control in TD, which remain to be explored.

Previous research reported inconclusive findings regarding voluntary motor control in TD. Studies in adults often revealed deficits in reflex inhibition and motor set selection or switching;^19-26^ (but see also references^23-26^ for divergent findings). In contrast, research on paediatric patients typically found no significant deficits^27-36^ or even better motor performance.^15,37,38^ Further, neuroimaging studies stress divergent findings between children and adults with TD about brain structure, activation patterns, and connectivity.^18,39-41^

Although voluntary motor performance of younger TD patients often resembles that of healthy controls (HC), it remains unclear whether comparable behaviour results from similar physiological motor control or rather reflects successful neural adaptations in these TD patients. Notably, the distinction between compensatory and dysfunctional adaptations has significant clinical implications. While compensatory mechanisms may support motor development, dysfunctional changes might perpetuate tics or impair broader motor control. Thus, clarifying these relationships is critical for understanding pathophysiological processes in TD and tailoring novel therapeutic interventions. For example, compensatory mechanisms that improve motor network development might be leveraged for therapeutic purposes, while identifying maladaptive processes could inform strategies to prevent long-term deficits. Of note, while adult TD patients often have a long-standing history of pharmacological treatments—including anti-dopaminergic drugs, central adrenergic inhibitors, SSRIs, or anti-epileptic medications—that can influence brain network development and organization, paediatric drug-naïve patients offer a unique opportunity to examine ‘natural’ neural network adaptation. In children, compensatory processes appear to involve heightened activity in frontal motor control regions, such as the prefrontal cortex (PFC) and supplementary motor area (SMA), with additional recruitment of ipsilateral motor areas.^7,14,31,42-46^ These findings suggest that motor performance in paediatric TD is supported by active reorganization of motor networks. However, whether these patterns reflect accelerated maturation or deviant development remains unclear.^16-18,31,40,41,47-51^ Understanding how brain motor networks (re-)organize during typical development and comparing paediatric TD patients to age-matched healthy controls could provide a developmental framework essential for identifying TD-specific adaptations and clarifying the pathophysiological processes underlying TD.

The current study addresses the gaps mentioned above by investigating neural alterations in paediatric drug-naïve TD and their associations with motor control. We integrated findings from age-matched healthy controls and a large cohort of typically developing children to contextualize TD-specific patterns within normative development. Using functional MRI (fMRI) and dynamic causal modelling (DCM), we examined neural activity and effective connectivity during reactive, goal-oriented movements assessed through a reaction time (RT) task. Behavioural measures of inhibitory control, evaluated using a blink-suppression paradigm, complemented the analyses.

We hypothesized that (i) motor performance in drug-naïve TD patients resembles or surpasses that of healthy controls, reflecting compensatory mechanisms; (ii) compensatory processes manifest as increased activation in motor regions and enhanced effective connectivity, particularly in frontal networks; and (iii) these neural patterns align with either normative developmental changes (accelerated maturation) or deviant adaptations specific to TD. Further elucidating these mechanisms may provide the foundation for targeted therapeutic approaches that harness beneficial compensatory changes and prevent maladaptive processes.

## Materials and methods

### Participants

The study involved 55 typically developing children and adolescents aged 5–17 years (*M*_age_ = 10.9, SD = 3.1, 46% male) and 21 never medicated patients aged 7–16 years (*M*_age_ = 10.2, SD = 2.3, 76% male), meeting DSM-V criteria for chronic Motor Tic Disorder (CTD; *N* = 2) or Tourette Syndrome (TS; *N* = 19). Table 1 provides an overview of the demographic and psychometric characteristics of the patient group and matched control sample included in the comparative behavioural analysis. Behavioural analyses of healthy subjects alone included sample sizes of *N* = 52 (task condition ‘Internal’) and *N* = 55 (task condition ‘External’), while fMRI analyses excluded some patients (*N*_Internal_ = 7; *N*_External_ = 9) and controls (*N*_Internal_ = 7; *N*_External_ = 12) due to excessive head motion. Further exclusions for DCM analysis occurred due to insufficient voxel response in ipsilateral regions of interest (*N*_patients_ = 2; *N*_controls_ = 19). Developmental samples retained broad age coverage despite motion-related exclusions, which were more common in younger participants. Detailed sample sizes for each paradigm and analysis type are provided in Supplementary Table 1; age distributions across developmental samples are summarized in Supplementary Table 2. Group comparability between patients and matched controls was ensured for all analyses through statistical comparisons of demographic and psychometric variables across subsamples. Exclusion criteria were (i) full-scale IQ < 70 (ii) history of epilepsy or other CNS disorders, (iii) significant premature birth (≤31 weeks), (iv) non-correctable visual impairments, (v) any contraindications to the MRI or (vi) current or previous use of psychoactive drugs [except for history of stimulant medication (*n* = 1)]. The Ethics Committee of the Medical Faculty, University Hospital Cologne approved the study, which adhered to the Declaration of Helsinki. Participants and parents provided informed assent and consent, respectively, and subjects received financial compensation.

**Table 1:** Demographic and psychometric characteristics of the patient and matched control sample

Sample characteristics	Controls (*N* = 20)	TD (*N* = 21)	Statistic	*P*
Age (M ± SD, range)	9.8 (±2.3; 6–15)	10.2 (±2.3; 7–16)	t (39) = 0.473	0.639
Male sex [*n* (%)]	15 (75)	16 (76)	χ^2^ (1) = 0.008	0.929
Right handedness [*n* (%)]	19 (95)	20 (95)	χ^2^ (1) = 0.001	0.972
IQ (M ± SD)	110.8 (±13.5)	108.1 (±14.9)	t (39) = −0.598	0.553
Working memory Index (M ± SD)	11.9 (±2.6)	11.7 (±3.2)	t (37) = −0.185	0.854
Processing speed Index (M ± SD)	10.1 (±1.9)	10.0 (±3.0)	t (36) = −0.070	0.944
DIKJ total symptom score (M ± SD)	8.9 (±4.8)	8.2 (±5.5)	t (37) = −0.481	0.633
YGTSS global severity score (M ± SD)		32 (±14.0)		
Age at tic onset (M ± SD)		4.9 (±1.8)		
Duration of tics [years (M ± SD; range)]		5.0 (±2.5; 1–9)		
Tourette syndrome [*n* (%)]		19 (90)		
Comorbid ADHD [*n* (%)]		5 (24)		

Handedness as assessed by the Edinburgh Handedness Scale. General IQ based on Wechsler Intelligence Scale for Children—5th Edition (WISC-V). Working memory, as assessed by Digit Span subtest (Scaled Scores/or Raw scores). Processing speed [i.e. visuomotor coordination (VMC)], as assessed by Coding subtest (Scaled Scores/or Raw scores). DIKJ = revised German version of Children's Depression Inventory; YGTSS = Yale Global Tic Severity Scale; M = mean; SD = standard deviation. Statistical comparisons of demographic and psychometric variables were conducted across all subsamples. No significant differences were found between groups in any of these characteristics.

### Measures

A structured clinical interview (Kinder-DIPS) was conducted with all parents and adolescents (aged ≥ 12 years), ensuring diagnostic criteria in patients and the absence of any neuropsychiatric symptoms in healthy controls. The interview is widely used in German-speaking clinical research and shows high interrater reliability (*k* = 0.78–0.95, depending on diagnostic category) for DSM-V-based psychiatric disorders.^52,53^ Full-scale IQ was assessed using the German adaptation of the Wechsler Intelligence Scale for Children—Fifth Edition (WISC-V), which shows excellent internal consistency (*α* = 0.96) and high convergent validity with other standard intelligence tests for children and adolescents (e.g. WPPSI-III: *r* = 0.89; KABC-II: *r* = 0.83). In addition to full-scale IQ, we extracted a Working Memory Index (Digit Span) and a Processing Speed Index (Coding), the latter reflecting visuomotor coordination (VMC).^54^ Tic severity was assessed using the Yale Global Tic Severity Scale (YGTSS), a clinician-rated instrument evaluating motor and vocal tics and their associated impairment.^55,56^ Depressive symptoms were measured using the revised German version of the Children's Depression Inventory (DIKJ).^57^ Handedness was assessed with the Edinburgh Handedness Inventory.^58^

### fMRI paradigm

**Figure 1:**
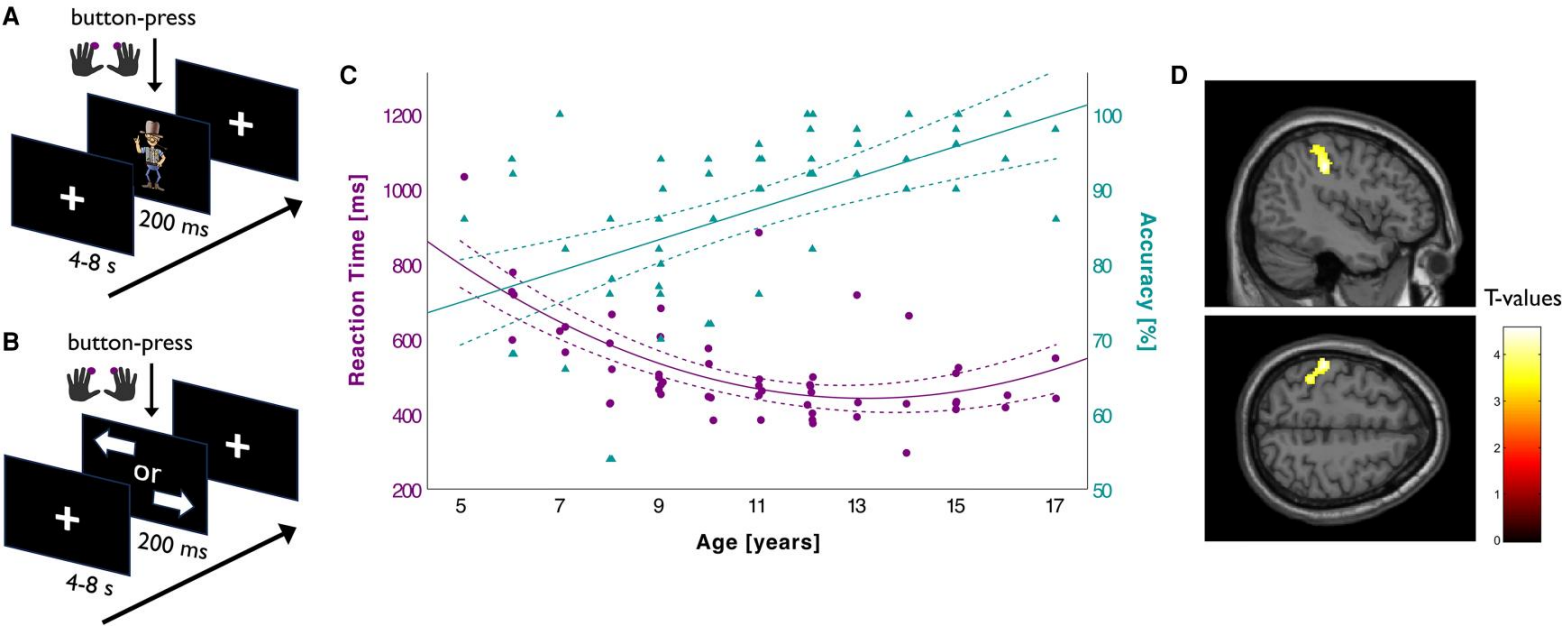
**Experimental paradigm and typical developmental trajectories.** (**A**) fMRI task condition ‘Internal’: Subjects were instructed to press either of two buttons as soon as possible following the appearance of a non-informative target-stimulus (Sherriff). (**B**) fMRI task condition ‘External’: Participants were instructed to press the button on the side indicated by an arrow as quickly as possible. (**C**) Developmental trajectory of externally cued responses in typically developing children and adolescents (*N* = 55). Regression analyses revealed linear age-related improvements in accuracy (triangles; *R*^2^ = 0.309, B = 0.021, *P* < 0.001) and a steep decline in reaction times during early childhood (circles; *R*^2^ = 0.251, B = −0.952, *P* < 0.001). Reaction times are displayed in milliseconds (ms). Each data point represents one participant. Dashed lines indicate 95% confidence intervals for the fitted regression lines. (**D**) Linear age-related increase in BOLD activation for externally cued right-handed movements in typically developing children and adolescents (*N* = 43), observed in a left-sided (contralateral) cluster including primary somatosensory cortex (S1), intraparietal sulcus (IPS), inferior parietal lobule and superior parietal lobule (SPL). Peak activation was located at MNI coordinates x = −44, y = −30, z = 40 (k = 427 voxels, t = 4.57). Activations are rendered on a canonical brain (P_FWE-corr._ < 0.001). Colour bar indicates t-values.

We aimed to investigate network-level mechanisms of voluntary motor control using a reaction time paradigm previously employed to study movement preparation, selection and initiation in healthy adults and neuropsychiatric conditions.^59-62^ To accommodate young children, the original blocked design was modified into an event-related format with two conditions presented separately (in randomized order). In the ‘Internal’ condition (Fig. 1A), subjects were instructed to press either of two buttons as soon as possible following the appearance of a non-informative target-stimulus (Sherriff). In this condition, subjects were free about the lateralization of their movement (left or right) but were restricted concerning response timing. In the ‘External’ condition (see Fig. 1B), participants were instructed to press the button on the side indicated by an arrow as quickly as possible. Both conditions ended after the participants completed 25 left- and right-handed button presses, respectively. Task stimuli were generated using the software package *Presentation* (Version 10.3, Neurobehavioral Systems Inc., Albany, CA, USA), projected onto a screen at the rear of the scanner bore, and viewed during image acquisition via an individually adjusted mirror mounted on the head coil.

### Blink-suppression paradigm

We assessed inhibitory control via a blink-suppression task consisting of six 36-s blocks (three ‘suppress’ and three ‘release’ blocks). Participants suppressed eye-blinks or blinked freely while viewing videoclips of blinking individuals, with task instructions cued by a traffic light signal. Blinks were video recorded and analysed for suppression efficacy using the Behavioural Observation Research Interactive Software.^63^ The number of blinks occurring during the task was counted by two independent raters, with excellent interrater reliability (ICC = 0.93; 95% CI: 0.87–0.97; *P* < 0.001).

### Statistical analysis of behavioural data

Reaction times (RTs) and task accuracy were analysed as behavioural measures derived from the fMRI paradigm. RT was defined as the latency between stimulus onset and button press; accuracy reflected the percentage of correct responses per condition. Across all trials, RTs below 150 ms, exceeding 2000 ms and outliers beyond 3 SD from individual averages per condition were excluded.^60^ Blink suppression, derived from the blink-suppression paradigm, was quantified as the difference between blink counts in suppression and release conditions, normalized as a percentage reduction.

To study the development of motor network functions, we explored the relationship between age (mean-centred) and fMRI task measures (RT, accuracy) using regression analyses, including linear, quadratic and cubic models within the healthy sample. Group differences between TD patients and age-matched healthy controls were calculated using independent-samples *t*-test or, in cases of non-normality (assessed via Shapiro–Wilk tests and histogram inspection), non-parametric Mann–Whitney U-tests. This approach was chosen due to the matched between-group design with relatively small group sizes, which limited the use of covariate-adjusted regression models. For more details, see Supplementary material.

### fMRI data acquisition and analyses

Participants were trained in a mock-scanner before the scanning session to minimize movement artefacts. They received feedback on head motion while practising the fMRI paradigms in a realistic setting. Additionally, participants’ heads were fixated using foam pads surrounding the head.

MRI scans were performed on a 3-Tesla Siemens MAGNETOM Prisma scanner (Siemens Healthcare, Erlangen, Germany) at Research Centre Juelich. T1-weighted structural images were acquired by a magnetization-prepared rapid gradient echo (MP-RAGE) sequence (repetition time [*T_R_*] = 1790 ms, echo time [*T_E_*] = 2.53 ms, flip angle = 8°, number of slices = 176, slice thickness = 0.9 mm, interslice gap = 0.45 mm, field of view [FOV] = 256 mm, voxel size = 0.9× 0.9× 0.9 mm). Whole-brain T2-weighted functional images were obtained using an echoplanar imaging multiband sequence, with blood oxygenation level-dependent (BOLD) contrast (*T_R_* = 980 ms, *T_E_* = 30 ms, flip angle = 70°, number of slices = 64, slice thickness = 2.0 mm, interslice gap = 0.2 mm, FOV = 207 mm, voxel size = 2.2× 2.2× 2.0 mm). Image pre-processing was performed using Statistical Parametric Mapping [SPM12; The Wellcome Centre for Human Neuroimaging, UCL Queen Square Institute of Neurology, London, UK (www.fil.ion.ucl.ac.uk/spm)] implemented in MATLAB (The MathWorks, Natick, USA). Pre-processing included motion correction, spatial normalization, and smoothing with an 8 mm Gaussian kernel. Detailed pre-processing steps are described in the Supplementary material. Head motion was assessed through visual inspection of motion plots generated by SPM12. Datasets with displacement exceeding one voxel (2.2 mm) or abrupt motion peaks greater than 1.1 mm were excluded, following thresholds commonly used in developmental fMRI studies.^64,65^ Following pre-processing, all datasets were visually re-inspected to ensure pre-processing quality.

Task-related BOLD responses were modelled using the GLM framework, with contrasts capturing left- and right-handed movements (from stimulus presentation until button presses) relative to baseline. Contrast images were defined as follows: ‘right-handed movements > baseline’ and ‘left-handed movements > baseline’. Model parameter estimates and t-statistic images were submitted to second level group analyses. Baseline task activations for these contrasts, obtained through one-sample *t*-tests for patients and control subjects separately, are provided in Supplementary Tables 3–10. In healthy subjects, we examined the association between age and whole-brain activation from these contrasts using regression analysis, adding mean-centred age, mean-centred age-squared and mean-centred age cubed as covariates. Moreover, using an independent-samples *t*-test, we compared task-related activity from these contrasts between TD patients and healthy controls. Effects were considered significant if they exceeded a voxel-level threshold (*P* < 0.001, uncorrected), cluster-level corrected at *P*_FWE_ < 0.05.

### Dynamic causal modelling

DCM was applied to explore interhemispheric motor-network connectivity, following Michely *et al*.,^61^ who used the paradigm to examine age-related connectivity changes in healthy adults. The same regions of interest were used to assess neural interactions across developmental stages. We specified nine ROIs for the interhemispheric DCM model: (i) left PFC, (ii) right PFC, (iii) left PMC, (iv) right PMC, (v) SMA, (vi) left M1, (vii) right M1, (viii) left IPS, and (ix) right IPS. Time series were extracted from subject-specific coordinates defined in the ‘External’ condition. Within an 8-mm-radius sphere around the group peak coordinates, which were set as origin (see Supplementary Table 11), we located the nearest individual activation peak coordinates from each subject's first level GLM-analysis. We extracted the first eigenvariate of the individual BOLD time series. For extraction of time series, we employed a threshold of *P* < 0.05 (uncorrected). Following recommendations by Zeidman *et al*.^66^ for handling cases where ROIs showed no significant voxel response at this threshold, a stepwise lowering of the threshold was conducted in steps of 0.05, until a peak was discernible. Group-level mean ROI coordinates are also reported in Supplementary Table 12.

We defined (i) the endogenous connectivity matrix (DCM-A), representing connectivity independent of task-dependent modulation; (ii) external inputs to the PFC and IPS (DCM-C), assuming experimental inputs directly influencing these regions; and (iii) nine models exploring alternative hypotheses about modulatory changes in interregional connectivity driven by task demands (i.e. right-handed responses, DCM-B). Random-effects Bayesian model selection was applied to determine winning models for the developmental cohort and for the group of TD patients and age-matched controls. Winning models were established based on posterior evidence, ensuring an optimal balance between model complexity and generalizability.

We examined the association between age (mean-centred) and coupling estimates of the winning model using regression analyses with linear, quadratic and cubic models. Following recommendations by Dash *et al*.,^67^ we identified outliers using the interquartile range (IQR) and employed winsorising to reduce the impact of outliers in the models. Values larger than Q3 + 1.5 * IQR or smaller than Q1–1.5 * IQR were considered outliers. Any value above or below this cut-off was substituted with the value of that cut-off itself. To investigate differences in neural coupling between TD patients and healthy controls, CEs of the winning model were compared using independent-samples *t*-test and non-parametric Mann–Whitney U-test. All analyses were conducted separately for endogenous connections (DCM-A) and task-specific connectivity (DCM-B).

Explorative correlation analyses tested associations of brain network organization and motor control. To this end, behavioural measures [RT, accuracy, working memory (WM), visuomotor coordination (VMC)]—the latter two reflecting higher-order cognitive and sensorimotor processes relevant to task performance—were related to measures of task activation (mean beta values) or CEs significantly associated with age or TD (DCM-A and DCM-B). No alpha adjustment was applied due to the exploratory nature of this analysis.

## Results

### Behavioural task performance

Regression analysis showed that age was significantly associated with accuracy in typically developing children and adolescents for both internally and externally cued responses. For accuracy, age displayed a positive linear association with both conditions (‘External’: *R*^2^ = 0.309, *B* = 0.021, *P* < 0.001; see Fig. 1C; ‘Internal’: *R*^2^ = 0.240, *B* = 1.401, *P* < 0.001; see Supplementary Fig. 1). For RTs, there was a significant cubic association between age and externally cued responses (*R*^2^ = 0.251, *B* = −0.952, *P* < 0.001; see Fig. 1C) and a significant quadratic association with age for internally cued responses (*R*^2^ = 0.359, *B* = 3.475, *P* < 0.001; see Supplementary Fig. 1), with RTs decreasing throughout development in both conditions.

Between-group comparisons revealed significant differences in accuracy for internal and external cues, with TD patients showing higher accuracy than age-matched controls in both conditions. In contrast, RTs did not significantly differ between groups for either cue type. Both groups exhibited significantly longer RTs (patients: *Z* = −4.015, *P* < 0.001, *r* = −0.88; controls: Z = −3.385, *P* < 0.001, *r* = −0.82) and reduced accuracy (patients: Z = 2.739, *P* = 0.006, *r* = 0.60; controls: Z = 3.480, *P* < 0.001, *r* = 0.84) for externally cued responses than internally cued responses. There were no significant correlations between accuracy and RT across cue types (‘External’: *t_b_*  _patients_ = 0.255, *P* = 0.114; *t_b_*  _controls_ = 0.059, *P* = 0.720, and ‘Internal’: *t_b_*  _patients_ = −0.163, *P* = 0.341; *t_b_*  _controls_ = −0.107, *P* = 0.560).

Between-group comparisons further revealed significant differences in blink reduction, with healthy control subjects demonstrating greater ability to suppress blinks than TD patients (patients: Mdn = 69.00, IQR = 36.50; controls: Mdn = 79.00, IQR = 31.25; U = 80.000, Z = −2.417, *P* = 0.015, *r* = −0.41). No between-group differences were evident regarding WM (patients: M = 11.71, SD = 3.23; controls: M = 11.89, SD = 2.56; t(37) = −0.185, *P* = 0.854, Cohen's d = −0.062) or VMC (patients: Mdn = 10.00, IQR = 4.00; controls: Mdn = 10.00, IQR = 3.50; U = 167.000, Z = −0.344, *P* = 0.750, *r* = −0.056).

### Neural activation patterns

No significant association was observed between age and task-related neural activations for internally cued responses in typically developing children and adolescents. In contrast, for externally cued right-handed responses, a significant linear increase in task-related BOLD activation was found alongside increasing age in a cluster within the left (contralateral) parietal lobe, which included the postcentral gyrus (primary somatosensory cortex; Areas 3a, 3b, 1, 2), the intraparietal sulcus (IPS; Areas hIp1, hIP2, hIP3), the inferior parietal lobule (Areas PF, PFt) and superior parietal lobule (SPL; Area 7PC) (*x*, *y*, *z* = −44, −30, 40; *k_E_*  _=_ 427; *t* = 4.57; *P*_FWE-corr._ = 0.005; see Fig. 1D). Moreover, task activation in this cluster significantly positively correlated with task accuracy (*r* = 0.375, *P* = 0.013) and VMC (*r* = 0.440, *P* = 0.005).

**Figure 2:**
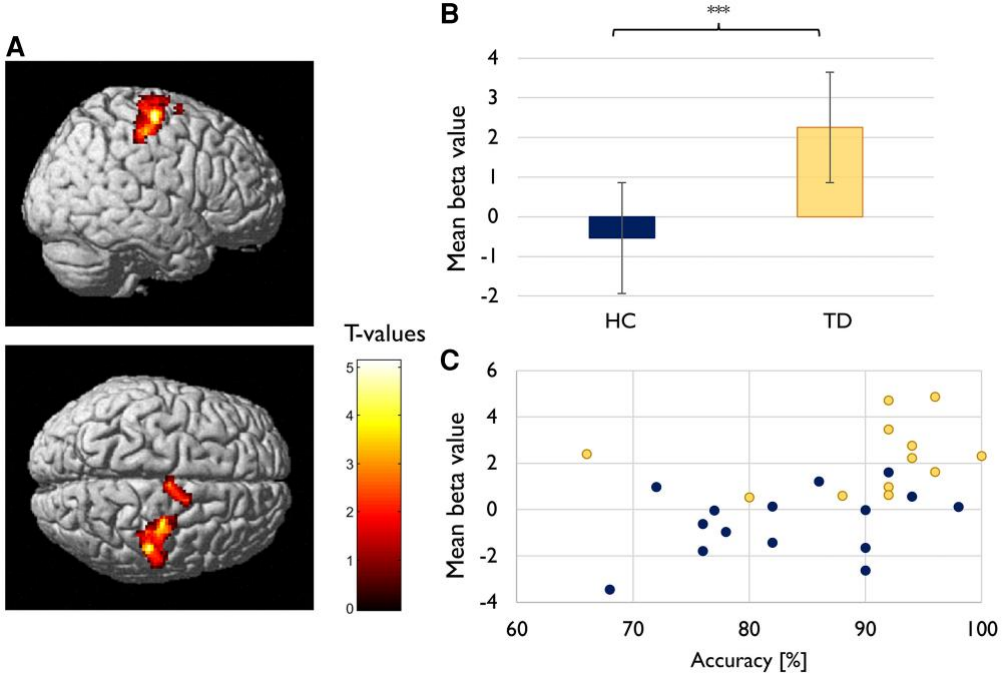
**Neural overactivation in patients with TD.** (**A**) Enhanced BOLD activation in TD patients (*N* = 12) compared to matched healthy controls (*N* = 15) during externally cued right-handed movements, observed in a right-sided (ipsilateral) cluster including the primary motor cortex (M1), primary somatosensory cortex (S1), and supplementary motor area (SMA). Activations are rendered on a canonical brain (*P*_FWE-corr._ < 0.001). Peak activation was located at MNI coordinates x = 36, y = −24, z = 74 (k = 845 voxels, t = 5.20). Colour bar indicates t-values. (**B**) Mean beta values derived from the peak voxel of this cluster. Between-group comparison (independent-samples *t*-test) revealed significantly higher activation in patients (*M* = 2.25, SD = 1.51) than in controls (*M* = −0.54, SD = 1.44), t(25) = 4.894, *** *P* < 0.001, two-tailed. Error bars represent the standard error of the mean. (**C**) Significant positive correlation between task accuracy and mean beta values from this cluster, using Kendall's tau-b (*t_b_* = 0.381, *P* = 0.006). Each data point represents one participant. In panels B and C, participant groups are distinguished by colour: healthy controls (HC) are shown in blue, and patients (TD) in yellow.

Between-group comparisons revealed no significant differences in task activation related to internally cued responses. In contrast, during externally cued right-handed movements, TD patients exhibited significantly enhanced activation in a right-sided (ipsilateral) cluster, including the precentral gyrus (primary motor cortex; Areas 4a, 4p), postcentral gyrus (primary somatosensory cortex; Areas 3a, 3b, 1, 2) and the supplementary motor area (SMA; Area 6d1) (*x*, *y*, *z* = 36, −24, 74; *k_E_*  _=_ 845; *t* = 5.20; *P*_FWE-corr._ < 0.001; see Fig. 2A and B). Additionally, beta-values derived from the peak voxel of this cluster significantly positively correlated with task accuracy (*t_b_* = 0.381, *P* = 0.006; see Fig. 2C).

### Connectivity analyses

#### Bayesian model selection

**Figure 3:**
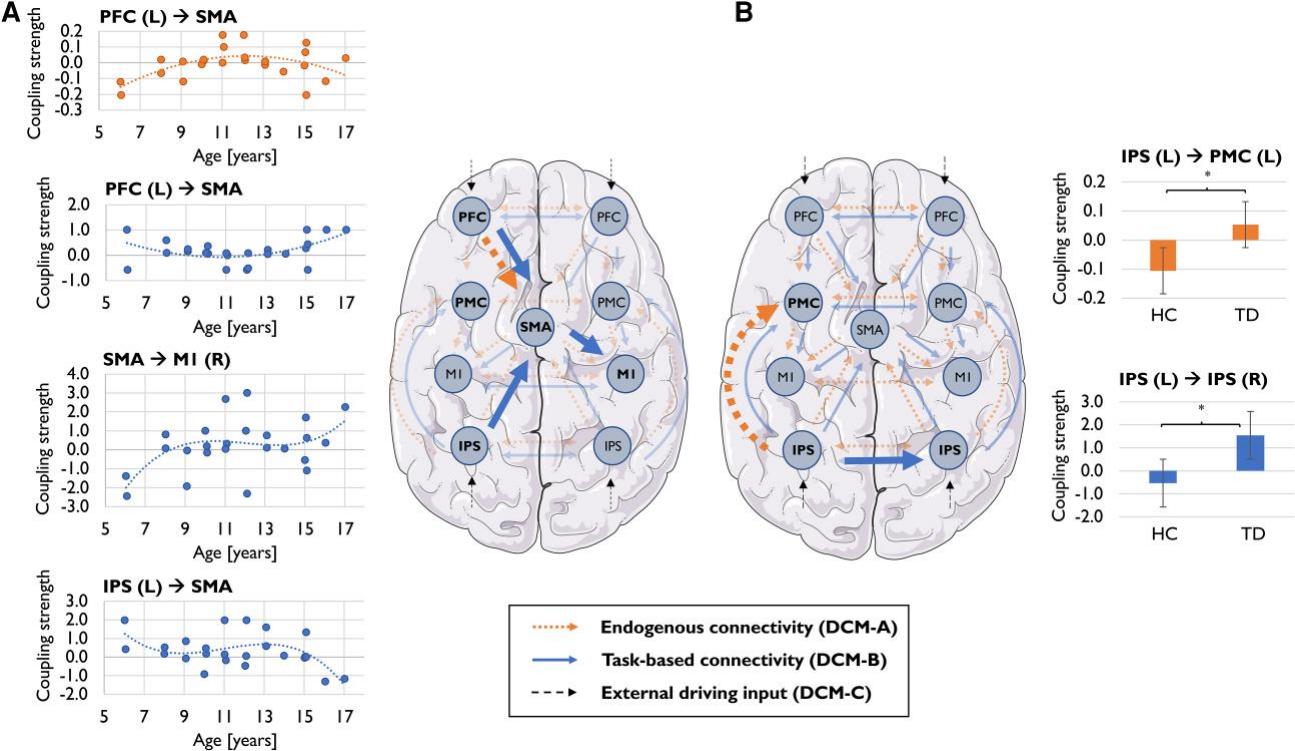
**Motor network connectivity in patients with TD and typically developing controls**. (**A**) Age-related changes in coupling strength in typically developing participants (*N* = 24). Each scatterplot depicts individual coupling estimates (y-axis) plotted against age (x-axis); dotted regression lines represent the best-fitting models. Regression analyses revealed a quadratic trajectory for endogenous PFC-SMA coupling (*R*^2^ = 0.285, *P* = 0.007), and for task-based PFC-SMA coupling (*R*^2^ = 0.209, *P* = 0.025), as well as cubic trajectories for IPS-SMA coupling (*R*^2^ = 0.226, *P* = 0.019) and SMA-M1 coupling (*R*^2^ = 0.247, *P* = 0.014). Each data point represents one participant. (**B**) Between-group differences in coupling estimates for endogenous (top) and task-based (bottom) connections. Patients (*N* = 10) showed significantly stronger excitatory connectivity from left IPS to left PMC (t(18) = 2.26, *P* = 0.036), and significantly enhanced excitatory task-based interhemispheric IPS-IPS coupling compared to controls (*N* = 10; U = 81.00, Z = 2.34, *P* = 0.019). Error bars represent the standard error of the mean. Asterisks denote statistically significant differences (*P* < 0.05, two-tailed). In both panels, connectivity types are distinguished by colour: endogenous connections (DCM-A) are shown in orange, task-based connections (DCM-B) in blue, and external driving input (DCM-C) in black. Bold arrows highlight connections with significant effects. L = left; R = right; HC = healthy controls; TD = patients; IPS = intraparietal sulcus; PMC = premotor cortex; PFC = prefrontal cortex; SMA = supplementary motor area; M1 = primary motor cortex.

According to Bayesian model selection, out of all interhemispheric models tested for the group of healthy controls, Model 4 (without interhemispheric PMC-coupling; Fig. 3A) was most likely given our data. Model selection across groups (patients and matched controls) revealed Model 5 (without interhemispheric M1-coupling; Fig. 3B) as the winning model. Supplementary Figs 2 and 3 provide a complete overview of the tested model space and the corresponding evidence supporting selection of the winning models.

#### Endogenous connectivity (DCM-A)

In typically developing children and adolescents, regression analysis revealed that CEs between the left PFC and the SMA followed a significant quadratic (inverted U-shaped) trajectory with age (*R*^2^ = 0.285, *B* = −0.005, *P* = 0.007; see Fig. 3A) and were significantly positively correlated with WM (*r* = 0.479, *P* = 0.028).

Significant deviations in endogenous connectivity were found in TD patients compared to age-matched controls using independent-samples *t*-tests. Notably, the left IPS in TD patients exerted an excitatory influence on the left PMC (*M* = 0.05, SD = 0.18), whereas this connection was inhibitory in healthy controls (*M* = −0.11, SD = 0.13, t(18) = 2.262, *P* = 0.036, Cohen's *d* = 1.012; see Fig. 3B). No significant correlation was found between the coupling strengths of this connection and measures of task performance, WM or VMC.

#### Task-dependent connectivity (DCM-B)

For interhemispheric task-dependent connections, regression analyses revealed that in healthy subjects, the connection from the left IPS to the SMA showed a significant cubic age-related decrease, transitioning from excitatory to inhibitory influence (*R*^2^ = 0.226, *B* = −0.006, *P* = 0.019; see Fig. 3A). Furthermore, CEs from this connection negatively correlated with WM (*t_b_* = −0.345, *P* = 0.033) and VMC (*t_b_* = −0.381, *P* = 0.017). Conversely, the connection from the SMA to the right M1 followed a cubic age-related shift from inhibitory to excitatory influence (*R*^2^ = 0.247, *B* = 0.010, *P* = 0.014; see Fig. 3A). Spearman's rank correlation further indicated a positive correlation between CEs of this connection and task accuracy (*r* = 0.407, *P* = 0.049). The excitatory influence from the left PFC to the SMA followed a quadratic (U-shaped) developmental pattern (*R*^2^ = 0.209, *B* = 0.023, *P* = 0.025; see Fig. 3A) and was not significantly correlated with any measure of task performance, WM or VMC.

Non-parametric between-group comparisons (Mann–Whitney U) revealed significant differences in task-dependent connectivity between patients with TD and controls. Specifically, the interhemispheric connection between the left and right IPS was excitatory in TD (Mdn = 0.59, IQR = 2.119) but inhibitory in controls (Mdn = 0.032, IQR = 0.554; U = 81.000, Z = 2.343, *P* = 0.019, *r* = 0.52; see Fig. 3B). Again, no significant correlation was found between CEs of this connection and measures of task performance, WM, or VMC. Supplementary Tables 13–15 provide group-mean coupling strengths for all examined connections, including both endogenous (DCM-A) and task-dependent (DCM-B) connectivity.

## Discussion

This study investigated neural mechanisms underlying motor control in paediatric drug-naïve TD patients, within the framework of typical motor development. TD patients outperformed healthy controls in task accuracy, suggesting enhanced reactive motor control, but exhibited deficits in sustained inhibitory control, as evidenced by impaired blink suppression. At the neural level, ipsilateral motor overactivation and altered interhemispheric connectivity patterns reflected TD-specific adaptations rather than delayed or accelerated normative trajectories. These results highlight a complex interplay between compensatory mechanisms that enhance reactive motor control and persistent deficits in inhibitory control, offering new insights into the pathophysiology of TD.

### Age-related motor development

Our developmental sample showed age-related improvements in motor performance, which were still evident in the age range between 5 and 16 years, consistent with previous research.^68-73^ In line with earlier studies in healthy adults,^60,61^ our child- and adolescent participants displayed longer RTs and reduced accuracy for directive cues compared to non-informative cues. This difference likely reflects the higher load in motor control required when participants not only had to ensure adequate timing but also choose the correct hand. In our sample, RTs declined steeply during early childhood and continued to improve more gradually into adolescence, showing a relative flattening of the curve after age 10 (see Fig. 1B). Accuracy improved in a more linear fashion, approaching adult-like performance by mid-adolescence.^61,69,74-77^ Comparisons with previous adult samples (aged 21–35)^61^ suggest that adolescents in our study (aged 13–16) still exhibited greater RT variability and overall slower average RTs. Furthermore, in the ‘Internal’ condition, slower RTs observed in our adolescent sample—relative to previously reported young adult data—may be attributed to the increased complexity of the task stimuli used in our study (i.e. pictures of a Sherriff versus double-sided arrows).

At the neural level, we observed an age-related linear increase in activation within a left parietal cluster, encompassing the inferior parietal lobule, IPS, SPL and postcentral gyrus. Similar age-related increases in parietal activation have previously been demonstrated in children and adolescents across a variety of motor- and WM tasks associated with attention, higher-order motor planning and response selection.^78-81^ While the parietal cortex is widely recognized for its involvement in visuospatial attention,^82,83^ specific regions are associated with distinct aspects of motor control, such as the storage of action representations (inferior parietal lobule),^84^ integration of visuospatial information into motor plans (IPS),^62,85^ online sensorimotor integration (SPL)^86^ and motor learning through somatosensory feedback (postcentral gyrus).^87,88^ In our sample, increased parietal activation was associated with enhanced task accuracy and VMC. Conversely, reduced activation in the left parietal and postcentral cortices has been linked to impaired motor performance in children with a developmental coordination disorder, emphasizing these regions’ significance in the development of integratory processes essential for motor refinement and coordination.^89^

### Motor adaptations in TD: enhanced reactive control versus challenges in sustained inhibition

Patients with TD outperformed healthy controls regarding task accuracy, while maintaining comparable RTs. This finding suggests that the ability to control cued volitional movements may be enhanced in children and adolescents with TD and aligns with a few studies showing improved motor performance in young TD patients.^14,15,37,38^ These improvements have been hypothesized to reflect compensatory processes driven by frequent inhibitory training through tic suppression.^15,43^ However, despite enhanced performance in the RT task, patients in our sample displayed deficits in sustained inhibitory motor control, as evidenced by impaired blink suppression (see Table 2). These findings challenge the assumption that compensatory mechanisms in TD are facilitated via training-induced increases in inhibition. Instead, these findings point to task-specific adaptations that selectively support reactive motor control. This distinction emphasizes the complexity of motor control in TD, where adaptations may be tailored to specific motor demands unrelated to tic suppression per se.

**Table 2:** Task performance parameters in patients and matched controls

Performance parameters	Controls	TD	Statistic	*P*
**Reaction time (Internal)** [M; SD]	417.83; 105.58	395.52; 77.35	t (36) = −0.752	0.457
**Accuracy (Internal)** [Mdn; IQR]	**0.95; 0.06**	**1.0; 0.05**	**Z = 2.361**	**0.021**
**Reaction time (External)** [Mdn; IQR]	482.0; 140.9	508.73; 182.1	Z = 0.026	0.979
**Accuracy (External)** [Mdn; IQR]	**0.80; 0.18**	**0.92; 0.15**	**Z = 1.999**	**0.046**
**Blink reduction** [Mdn; IQR]	**79.00; 31.25**	**69.00; 36.50**	**Z = −2.417**	**0.015**

Bold values indicate statistically significant results (*P* < 0.05). M = mean; SD = standard deviation; Mdn = median; IQR = interquartile range.

### Neural overactivation as a compensatory mechanism?

Improved motor performance in patients with TD was linked to distinct changes in motor network activation patterns: for right-handed movements, patients exhibited ipsilateral overactivation of M1 and S1, which correlated positively with task accuracy. These findings align with previous research showing that children with TD recruit additional brain networks during voluntary movements compared to healthy controls.^45,46,90^ While earlier studies proposed that recruitment of additional motor resources may reflect compensatory mechanisms, they lacked performance measures to relate neural activity to behaviour. Conversely, our current findings directly link altered motor network activity and enhanced reactive motor control in young TD patients. Interestingly, this compensatory pattern contrasts with earlier findings in adult TD patients, where reduced task-related activation in primary and secondary motor cortices has been linked to poorer performance, suggesting that such compensatory mechanisms may be impaired or insufficiently developed in adult TD patients.^22^

Unlike dysfunctional overactivations seen in older adults or patients with early-onset neurodegenerative diseases (e.g. Huntington's, Alzheimer's and Parkinson’s), the ipsilateral overactivations in our TD sample appeared highly efficient, likely enhancing performance rather than merely compensating for deficits.^61,91-97^ In children with TD, prior studies have reported decreased activation in contralateral motor regions during voluntary movements, paired with increased frontal activations.^45,46,90^ These reductions may reflect top-down inhibition of contralateral motor areas, which have been demonstrated to be hyper-excitable at rest^98,99^ and modulated before volitional movements.^9,14,18,27,28^ Our data did not indicate increased frontal influence or reduced engagement of contralateral motor regions. Instead, ipsilateral overactivation may represent an alternative compensatory strategy, enhancing task performance by recruiting additional resources. Although these compensatory adaptations appear highly efficient, they seem specifically beneficial for reactive motor control. The observed deficits in sustained inhibition, such as impaired blink suppression, suggest that this adaptation may not generalize across different domains of motor control. This raises two possibilities: first, that the compensatory mechanisms enhancing reactive control may be task-specific and operate independently of inhibitory ability, or second, that these adaptations cannot efficiently counteract deficits in sustained inhibitory control. These findings emphasize the complexity of compensatory processes and highlight the importance of considering task-specific demands when evaluating TD motor adaptations. Notably, prior research suggests substantial heterogeneity within the TD population. For instance, Tajik-Parvinchi and Sandor^100^ reported that while some children with TD may develop adaptive mechanisms to control their tics—leading to improved voluntary control over eye movements—others exhibit reduced saccadic inhibitory control.

### Interhemispheric connectivity and motor networks in TD

Consistent with our task activation findings, effective PFC connectivity to motor regions was not significantly altered. Rather, we identified abnormal interhemispheric connectivity in patients with TD, which has previously been reported in both adult and paediatric TD and has mainly been associated with reduced interhemispheric inhibition.^41,49,101-103^

In our sample, patients with TD displayed increased excitatory task-based connectivity from left to right IPS. Given the IPS’ prominent role in integrating spatial information and coordinating attentional resources for movement planning,^62,85^ this may reflect altered functional integration of somatosensory information across hemispheres, which may help maintain control over motor outputs.

Within the left hemisphere, we observed significant between-group differences in endogenous connectivity from the IPS to the PMC. In healthy controls, the IPS exerted an inhibitory influence on PMC, while in TD patients, this influence was excitatory. This shift from inhibition to excitation could contribute to the hyperexcitability of contralateral motor regions commonly observed in TD patients.^98,99^ Alternatively, it may reflect stronger interactions between perceptual and motor processes, as suggested by increased perception-action binding previously documented in TD patients.^104-106^ Despite these alterations, connectivity changes did not significantly correlate with behavioural performance, suggesting they represent broader network adaptations rather than direct compensatory mechanisms. Future research should investigate whether these patterns reflect pathological adaptive processes and their relationship to symptom severity and disease chronicity.

### Typical motor development: effective connectivity changes with age

In healthy subjects, age-related increases in activation within the left parietal lobe were accompanied by changes in effective connectivity linked to task accuracy, WM and VMC. These changes prominently involved the SMA, a region critically involved in initiating and coordinating voluntary movements. The SMA is known to play a dual role in facilitating intended actions while suppressing unintended ones, positioning it as a central hub in motor planning and execution networks.^107-112^ Age-related connectivity changes in SMA-associated pathways suggest that the SMA plays a critical role in supporting healthy motor development, integrating signals from other brain regions to enhance motor control over time. Age-related shifts in SMA connectivity suggest a developmental transition from reactive to proactive motor control, aligning with previous findings.^73,74^ Specifically, our data showed a change in the SMA's influence on ipsilateral M1, evolving from inhibitory in younger children to excitatory in older children. This influence positively correlated with task accuracy, suggesting that excitatory SMA-M1 coupling supports more precise motor execution throughout development. Connections from the left IPS to the SMA also displayed age-related changes, following a reverse trajectory to the SMA-M1 connection. This connectivity shift, in turn, was associated with measures of WM and VMC, where inhibitory input from IPS to SMA was linked to higher performance on both Digit Span and Coding subtests. These findings suggest that the IPS plays a critical role in modulating SMA activity to balance motor output with cognitive demands.

PFC connections to the SMA exhibited distinct developmental patterns for endogenous and task-based connectivity, which followed quadratic trajectories in opposite directions, with connectivity patterns in the youngest and oldest subjects appearing comparable. Notably, endogenous PFC-SMA connectivity positively correlated with WM, highlighting the PFC's role in supporting higher-order cognitive processes during motor performance.^79,80,113^

### Developmental trajectories in TD: deviant, delayed or accelerated?

Our findings offer a nuanced picture of developmental trajectories in TD. Behaviourally, patients outperformed age-matched controls in task accuracy while exhibiting comparable RTs, consistent with previous reports of enhanced reactive motor control and possibly indicating accelerated motor development.^14,15,37^ However, their deficits in sustained voluntary motor control may point toward delayed motor development in this domain, which was previously suggested to improve steadily throughout middle childhood and reach mature levels by early adolescence.^114^

At the neural level, activation patterns in TD patients deviated from the age-related changes seen in healthy controls. While accuracy improvements in controls were associated with increased task-related activation in the contralateral parietal cortex, enhanced accuracy in patients was linked to pronounced activation in ipsilateral M1 and S1. Notably, ipsilateral overactivation partially mirrored the contralateral activations observed in typically developing children, as both involved the primary somatosensory cortex. This observation implies that ipsilateral overactivation serves as a compensatory mechanism, deviating from typical developmental trajectories yet enhancing reactive motor control and potentially mitigating deficits associated with TD.^7-12^

Similarly, task-related connectivity in patients with TD diverged from age-related changes in healthy controls. Interestingly, intra-, and interhemispheric connectivity alterations in TD patients involved the left IPS, a region showing age-related activation increases in typically developing children and adolescents. While this indicates increased parietal cortex engagement in TD patients, mirroring recruitment patterns in older children, other findings, e.g. increased excitatory endogenous connectivity from the IPS to the PMC, may reflect pathological rather than compensatory mechanisms. This altered connectivity could contribute to hyperexcitability of motor regions or enhanced action-perception binding, both commonly reported in TD.^98,99,104-106^ However, the lack of significant correlations between connectivity measures and behaviour renders a conclusive distinction of compensation and pathophysiological processes highly challenging and modulatory approaches are needed to further explore the mechanistic role of these connectivity changes in the future.

In conclusion, early deficits in TD may drive compensatory changes that resemble typical or even accelerated development on the behavioural level. Conversely, when compensatory processes are insufficient, pathological neural dynamics may lead to behavioural deficits comparable to delayed development. At the neural level, compensatory mechanisms produce developmental trajectories distinct from typical patterns, reflecting a dynamic interplay between adaptation and pathophysiological processes.^40,43^

## Limitations

While our current study advances the understanding of typical motor development and compensatory mechanisms in TD, several limitations must be addressed: (i) Small sample sizes necessitate caution in interpreting our findings, which require replication in larger cohorts to enhance robustness and generalisability. Significant age- and TD-related activation patterns were identified, but only when using a more lenient voxel-level threshold. Our focus on right-handed responses required excluding participants with insufficient ipsilateral task activation from DCM analysis. This reduced sample size but allowed for integrating behavioural, activation and connectivity analyses, revealing significant group- and age-related effects on both endogenous and task-based motor-network connectivity. (ii) While our cross-sectional design allowed for contextualization within a developmental framework, future longitudinal studies are essential to capture the dynamic nature of developmental trajectories in TD and further investigate compensatory mechanisms, particularly about symptom remission versus persistence in adulthood. (iii) Including patients with comorbid ADHD limits the attribution of findings exclusively to TD but enhances ecological validity by reflecting the clinical reality of high comorbidity rates in paediatric TD.^115^

## Conclusion

Our study highlights the dual nature of motor adaptations in children and adolescents with TD, combining a developmental and clinical approach. Enhanced accuracy in reactive motor tasks was supported by compensatory overactivation of ipsilateral motor regions and altered interhemispheric connectivity, likely reflecting efficient neural adaptations tailored to specific task demands. However, deficits in sustained inhibition, such as impaired blink suppression, suggest that these compensatory mechanisms do not generalize across all motor domains.

In typically developing children, age-related increases in parietal activation and SMA connectivity were associated with improved motor precision, WM and VMC. These findings provide insights into the typical developmental trajectory of motor networks and the role of integrative brain regions in motor control.

In contrast, TD patients exhibited patterns of neural activation and effective connectivity that diverged from typical development, reflecting TD-specific adaptations rather than delayed or accelerated maturation. These adaptations highlight the interplay between compensation and pathophysiological processes in TD, where efficient reactive control coexists with persistent deficits in inhibitory control.

Future research should explore how these adaptations evolve with age and whether they contribute to long-term symptom management or persistence. Understanding the balance between adaptive and maladaptive processes in TD could inform targeted interventions to enhance compensatory mechanisms while mitigating pathological changes.

## Supplementary Material

fcaf260_Supplementary_Data

## Data Availability

The data and project-specific code supporting the findings of this study are archived in the CRC1451 data registry at https://www.crc1451.uni-koeln.de/. This includes batch scripts for standard pre-processing and model estimation pipelines, generated via the graphical user interface in SPM12 [Wellcome Centre for Human Neuroimaging, UCL Queen Square Institute of Neurology, London, UK (www.fil.ion.ucl.ac.uk/spm)], as well as an adapted batch script for dynamic causal modelling, based on code available at https://www.fil.ion.ucl.ac.uk/spm/data/attention/. Access can be granted upon reasonable request via the CRC1451 registry.

## References

[1] Black KJ, Black ER, Greene DJ, Schlaggar BL. Provisional Tic disorder: What to tell parents when their child first starts ticcing. F1000Res. 2016;5:696. 27158458 10.12688/f1000research.8428.1 PMC4850871

[2] Cohen SC, Leckman JF, Bloch MH. Clinical assessment of Tourette syndrome and tic disorders. Neurosci Biobehav Rev. 2013;37(6):997–1007. 23206664 10.1016/j.neubiorev.2012.11.013 PMC3674220

[3] Müller-Vahl K . Tourette-Syndrom und andere Tic-Erkrankungen im Kindes- und Erwachsenenalter. Klinische Neurophysiologie. 2015;46(02):109–109.

[4] Spencer T, Biederman J, Harding M, Wilens T, Faraone S. The relationship between tic disorders and Tourette's syndrome revisited. J Am Acad Child Adolesc Psychiatry. 1995;34(9):1133–1139. 7559306 10.1097/00004583-199509000-00009

[5] Tourette Association of America . Spectrum of Tourette Syndrome and Tic Disorders: Consensus of the Scientific Advisors of the Tourette Association of America. Accessed 11 December 2024. https://tourette.org/spectrum-tourette-syndrome-tic-disorders-consensus-scientific-advisors-tourette-association-america/

[6] Knight T, Steeves T, Day L, Lowerison M, Jette N, Pringsheim T. Prevalence of tic disorders: A systematic review and meta-analysis. Pediatr Neurol. 2012;47(2):77–90. 22759682 10.1016/j.pediatrneurol.2012.05.002

[7] Debes N, Preel M, Skov L. Functional neuroimaging in Tourette syndrome: Recent perspectives. Neurosci Neuroecon. 2017;6:1–13.

[8] Franzkowiak S, Pollok B, Biermann-Ruben K, et al Motor-cortical interaction in Gilles de la Tourette syndrome. PLoS One. 2012;7(1):e27850. 22238571 10.1371/journal.pone.0027850 PMC3251574

[9] Heise KF, Steven B, Liuzzi G, et al Altered modulation of intracortical excitability during movement preparation in Gilles de la Tourette syndrome. Brain. 2010;133(Pt 2):580–590. 20008030 10.1093/brain/awp299

[10] Jackson GM, Draper A, Dyke K, Pépés SE, Inhibition JS. Disinhibition, and the control of action in Tourette Syndrome. Trends Cogn Sci. 2015;19(11):655–665. 26440120 10.1016/j.tics.2015.08.006

[11] Rae C, Critchley H. Mechanistic insight into the pathophysiological basis of Tourette syndrome. Int Rev Mov Disord. 2022;3:209–244.

[12] Worbe Y, Marrakchi-Kacem L, Lecomte S, et al Altered structural connectivity of cortico-striato-pallido-thalamic networks in Gilles de la Tourette syndrome. Brain. 2015;138(Pt 2):472–482. 25392196 10.1093/brain/awu311 PMC4306818

[13] Mink JW . The basal ganglia and involuntary movements: Impaired inhibition of competing motor patterns. Arch Neurol. 2003;60(10):1365–1368. 14568805 10.1001/archneur.60.10.1365

[14] Jackson SR, Parkinson A, Jung J, et al Compensatory neural reorganization in Tourette syndrome. Curr Biol. 2011;21(7):580–585. 21439830 10.1016/j.cub.2011.02.047 PMC3076629

[15] Mueller SC, Jackson GM, Dhalla R, Datsopoulos S, Hollis CP. Enhanced cognitive control in young people with Tourette's syndrome. Curr Biol. 2006;16(6):570–573. 16546080 10.1016/j.cub.2006.01.064

[16] Plessen KJ, Bansal R, Peterson BS. Imaging evidence for anatomical disturbances and neuroplastic compensation in persons with Tourette syndrome. J Psychosom Res. 2009;67(6):559–573. 19913660 10.1016/j.jpsychores.2009.07.005 PMC4283588

[17] Church JA, Wenger KK, Dosenbach NU, Miezin FM, Petersen SE, Schlaggar BL. Task control signals in pediatric Tourette syndrome show evidence of immature and anomalous functional activity. Front Hum Neurosci. 2009;3:38. 19949483 10.3389/neuro.09.038.2009 PMC2784679

[18] Pépés SE, Draper A, Jackson GM, Jackson SR. Effects of age on motor excitability measures from children and adolescents with Tourette syndrome. Dev Cogn Neurosci. 2016;19:78–86. 26934638 10.1016/j.dcn.2016.02.005 PMC6988104

[19] Channon S, Drury H, Martinos M, Robertson MM, Orth M, Crawford S. Tourette's syndrome (TS): Inhibitory performance in adults with uncomplicated TS. Neuropsychology. 2009;23(3):359–366. 19413449 10.1037/a0014552

[20] Dursun SM, Burke JG, Reveley MA. Antisaccade eye movement abnormalities in Tourette syndrome: Evidence for cortico-striatal network dysfunction? J Psychopharmacol. 2000;14(1):37–39. 10757251 10.1177/026988110001400104

[21] Georgiou N, Bradshaw JL, Phillips JG, Bradshaw JA, Chiu E. The Simon effect and attention deficits in Gilles de la Tourette's syndrome and Huntington's disease. Brain. 1995;118(Pt 5):1305–1318. 7496788 10.1093/brain/118.5.1305

[22] Thomalla G, Jonas M, Baumer T, et al Costs of control: Decreased motor cortex engagement during a Go/NoGo task in Tourette's syndrome. Brain. 2014;137(Pt 1):122–136. 24176975 10.1093/brain/awt288

[23] Fan S, Cath DC, van der Werf YD, de Wit S, Veltman DJ, van den Heuvel OA. Trans-diagnostic comparison of response inhibition in Tourette's disorder and obsessive-compulsive disorder. World J Biol Psychiatry. 2018;19(7):527–537. 28741401 10.1080/15622975.2017.1347711

[24] Ganos C, Kuhn S, Kahl U, et al Action inhibition in Tourette syndrome. Mov Disord. 2014;29(12):1532–1538. 24995958 10.1002/mds.25944

[25] Rae CL, Parkinson J, Betka S, et al Amplified engagement of prefrontal cortex during control of voluntary action in Tourette syndrome. Brain Commun. 2020;2(2):fcaa199. 33409490 10.1093/braincomms/fcaa199 PMC7772099

[26] Rawji V, Modi S, Latorre A, et al Impaired automatic but intact volitional inhibition in primary tic disorders. Brain. 2020;143(3):906–919. 32125364 10.1093/brain/awaa024 PMC7089661

[27] Draper A, Stephenson MC, Jackson GM, et al Increased GABA contributes to enhanced control over motor excitability in Tourette syndrome. Curr Biol. 2014;24(19):2343–2347. 25264251 10.1016/j.cub.2014.08.038 PMC4188813

[28] Jackson SR, Parkinson A, Manfredi V, Millon G, Hollis C, Jackson GM. Motor excitability is reduced prior to voluntary movements in children and adolescents with Tourette syndrome. J Neuropsychol. 2013;7(1):29–44. 22804795 10.1111/j.1748-6653.2012.02033.x PMC3618371

[29] Jung J, Jackson SR, Parkinson A, Jackson GM. Cognitive control over motor output in Tourette syndrome. Neurosci Biobehav Rev. 2013;37(6):1016–1025. 23017869 10.1016/j.neubiorev.2012.08.009

[30] Jurgiel J, Miyakoshi M, Dillon A, Piacentini J, Makeig S, Loo SK. Inhibitory control in children with tic disorder: Aberrant fronto-parietal network activity and connectivity. Brain Commun. 2021;3(2):fcab067. 33977267 10.1093/braincomms/fcab067 PMC8093924

[31] Marsh R, Zhu H, Wang Z, Skudlarski P, Peterson BS. A developmental fMRI study of self-regulatory control in Tourette's syndrome. Am J Psychiatry. 2007;164(6):955–966. 17541057 10.1176/appi.ajp.164.6.955 PMC2291294

[32] Openneer TJC, van der Meer D, Marsman JC, et al Impaired response inhibition during a stop-signal task in children with Tourette syndrome is related to ADHD symptoms: A functional magnetic resonance imaging study. World J Biol Psychiatry. 2021;22(5):350–361. 32821008 10.1080/15622975.2020.1813329

[33] Raz A, Zhu H, Yu S, et al Neural substrates of self-regulatory control in children and adults with Tourette syndrome. Can J Psychiatry. 2009;54(9):579–588. 19751546 10.1177/070674370905400902 PMC4317356

[34] Roessner V, Albrecht B, Dechent P, Baudewig J, Rothenberger A. Normal response inhibition in boys with Tourette syndrome. Behav Brain Funct. 2008;4:29. 18638368 10.1186/1744-9081-4-29 PMC2491645

[35] Schmidgen J, Konrad K, Roessner V, Bender S. The external evocation and movement-related modulation of motor cortex inhibition in children and adolescents with Tourette syndrome—A TMS/EEG study. Front Neurosci. 2023;17:1209801. 37928740 10.3389/fnins.2023.1209801 PMC10620315

[36] Serrien DJ, Orth M, Evans AH, Lees AJ, Brown P. Motor inhibition in patients with Gilles de la Tourette syndrome: Functional activation patterns as revealed by EEG coherence. Brain. 2005;128(Pt 1):116–125. 15496435 10.1093/brain/awh318

[37] Jackson GM, Mueller SC, Hambleton K, Hollis CP. Enhanced cognitive control in Tourette Syndrome during task uncertainty. Exp Brain Res. 2007;182(3):357–364. 17569034 10.1007/s00221-007-0999-8

[38] Jung J, Jackson SR, Nam K, Hollis C, Jackson GM. Enhanced saccadic control in young people with Tourette syndrome despite slowed pro-saccades. J Neuropsychol. 2015;9(2):172–183. 24739098 10.1111/jnp.12044

[39] Gerard E, Peterson BS. Developmental processes and brain imaging studies in Tourette syndrome. J Psychosom Res. 2003;55(1):13–22. 12842227 10.1016/s0022-3999(02)00581-0

[40] Nielsen AN, Gratton C, Church JA, et al Atypical functional connectivity in Tourette syndrome differs between children and adults. Biol Psychiatry. 2020;87(2):164–173. 31472979 10.1016/j.biopsych.2019.06.021 PMC6925331

[41] Plessen KJ, Wentzel-Larsen T, Hugdahl K, et al Altered interhemispheric connectivity in individuals with Tourette's disorder. Am J Psychiatry. 2004;161(11):2028–2037. 15514403 10.1176/appi.ajp.161.11.2028

[42] Baym CL, Corbett BA, Wright SB, Bunge SA. Neural correlates of tic severity and cognitive control in children with Tourette syndrome. Brain. 2008;131(Pt 1):165–179. 18056159 10.1093/brain/awm278

[43] Eichele H, Plessen KJ. Neural plasticity in functional and anatomical MRI studies of children with Tourette syndrome. Behav Neurol. 2013;27(1):33–45. 23187150 10.3233/BEN-120294 PMC5213808

[44] Polyanska L, Critchley HD, Rae CL. Centrality of prefrontal and motor preparation cortices to Tourette syndrome revealed by meta-analysis of task-based neuroimaging studies. Neuroimage Clin. 2017;16:257–267. 28831377 10.1016/j.nicl.2017.08.004 PMC5554925

[45] Roessner V, Wittfoth M, August JM, Rothenberger A, Baudewig J, Dechent P. Finger tapping-related activation differences in treatment-naive pediatric Tourette syndrome: A comparison of the preferred and nonpreferred hand. J Child Psychol Psychiatry. 2013;54(3):273–279. 22774921 10.1111/j.1469-7610.2012.02584.x

[46] Roessner V, Wittfoth M, Schmidt-Samoa C, Rothenberger A, Dechent P, Baudewig J. Altered motor network recruitment during finger tapping in boys with Tourette syndrome. Hum Brain Mapp. 2012;33(3):666–675. 21391282 10.1002/hbm.21240 PMC6870320

[47] Debes N, Jeppesen S, Raghava JM, Groth C, Rostrup E, Skov L. Longitudinal magnetic resonance imaging (MRI) analysis of the developmental changes of Tourette syndrome reveal reduced diffusion in the cortico-striato-thalamo-cortical pathways. J Child Neurol. 2015;30(10):1315–1326. 25535056 10.1177/0883073814560629

[48] Greene DJ, Williams Iii AC, Koller JM, Schlaggar BL, Black KJ; The Tourette Association of America Neuroimaging Consortium. Brain structure in pediatric Tourette syndrome. Mol Psychiatry. 2017;22(7):972–980. 27777415 10.1038/mp.2016.194 PMC5405013

[49] Neuner I, Kupriyanova Y, Stocker T, et al White-matter abnormalities in Tourette syndrome extend beyond motor pathways. Neuroimage. 2010;51(3):1184–1193. 20188196 10.1016/j.neuroimage.2010.02.049

[50] Eichele H, Eichele T, Marquardt L, et al Development of performance and ERPs in a flanker task in children and adolescents with Tourette syndrome-A follow-up study. Front Neurosci. 2017;11:305. 28659750 10.3389/fnins.2017.00305 PMC5466959

[51] Makki MI, Govindan RM, Wilson BJ, Behen ME, Chugani HT. Altered fronto-striato-thalamic connectivity in children with Tourette syndrome assessed with diffusion tensor MRI and probabilistic fiber tracking. J Child Neurol. 2009;24(6):669–678. 19491113 10.1177/0883073808327838

[52] Schneider S, Pflug V, In-Albon T, Margraf J. Kinder-DIPS open access: Diagnostisches interview bei psychischen störungen im kindes- und jugendalter. 3rd ed. Mental Health Research and Treatment Center, Ruhr-University Bochum; 2018.

[53] Margraf J, Cwik JC, Pflug V, Schneider S. Strukturierte klinische Interviews zur Erfassung psychischer Störungen über die Lebensspanne: Gütekriterien und Weiterentwicklungen der DIPS-Verfahren. Z Klin Psychol Psychother. 2017;46(3):176–186.

[54] Wechsler D . Reliability and validity. Wechsler intelligence scale for children—Fifth edition (WISC-V): Technical and Interpretive Manual. Translated and adapted by Petermann F. Pearson Assessment; 2017:75–127.

[55] Leckman JF, Riddle MA, Hardin MT, et al The Yale global tic severity scale: Initial testing of clinician-rated scale of tic severity. J Am Acad Child and Adolesc Psychiatry. 1989;28(4):556–573. 10.1097/00004583-198907000-00015 2768151

[56] Storch EA, Murphy TK, Geffken GR, Sajid M, Allen P, Roberti JW. Reliability and validity of the Yale global tic severity scale. Psychol Assess. 2005;17:486–491. 16393016 10.1037/1040-3590.17.4.486

[57] Stiensmeier-Pelster J, Braune-Krickau M, Schürmann M, Duda K. Depressionsinventar für Kinder und Jugendliche (DIKJ). 3rd ed. Hogrefe; 2014.

[58] Oldfield RC . The assessment and analysis of handedness: The Edinburgh inventory. Neuropsychologia. 1971;9(1):97–113. 5146491 10.1016/0028-3932(71)90067-4

[59] Michely J, Barbe MT, Hoffstaedter F, et al Differential effects of dopaminergic medication on basic motor performance and executive functions in Parkinson's disease. Neuropsychologia. 2012;50(10):2506–2514. 22776611 10.1016/j.neuropsychologia.2012.06.023

[60] Michely J, Volz LJ, Barbe MT, et al Dopaminergic modulation of motor network dynamics in Parkinson's disease. Brain. 2015;138(Pt 3):664–678. 25567321 10.1093/brain/awu381 PMC4339773

[61] Michely J, Volz LJ, Hoffstaedter F, et al Network connectivity of motor control in the ageing brain. Neuroimage Clin. 2018;18:443–455. 29552486 10.1016/j.nicl.2018.02.001 PMC5852391

[62] Hoffstaedter F, Grefkes C, Zilles K, Eickhoff SB. The “what” and “when” of self-initiated movements. Cereb Cortex. 2013;23(3):520–530. 22414772 10.1093/cercor/bhr391 PMC3593700

[63] Friard O, Gamba M. BORIS: A free, versatile open-source event-logging software for video/audio coding and live observations. Methods Ecol Evol. 2016;7:1325–1330.

[64] Peelen MV, Glaser B, Vuilleumier P, Eliez S. Differential development of selectivity for faces and bodies in the fusiform gyrus. Dev Sci. 2009;12(6):F16–F25. 19840035 10.1111/j.1467-7687.2009.00916.x

[65] Walbrin J, Mihai I, Landsiedel J, Koldewyn K. Developmental changes in visual responses to social interactions. Dev Cogn Neurosci. 2020;42:100774. 32452460 10.1016/j.dcn.2020.100774 PMC7075793

[66] Zeidman P, Jafarian A, Corbin N, et al A guide to group effective connectivity analysis, part 1: First level analysis with DCM for fMRI. Neuroimage. 2019;200:174–190. 31226497 10.1016/j.neuroimage.2019.06.031 PMC6711459

[67] Dash C, Behera AK, Dehuri S, Ghosh A. An outliers detection and elimination framework in classification task of data mining. Decision Analytics Journal. 2023;6:100164.

[68] Bucsuházy K, Semela M. Case study: Reaction time of children according to age. Procedia Eng. 2017;187:408–413.

[69] Denckla MB . Development of motor co-ordination in normal children. Dev Med Child Neurol. 1974;16(6):729–741. 4442654 10.1111/j.1469-8749.1974.tb03393.x

[70] Hale S . A global developmental trend in cognitive processing speed. Child Dev. 1990;61(3):653–663. 2364741

[71] Haywood K, Getchell N. Lifespan motor development. 5th ed. Human Kinetics; 2009.

[72] Rueda MR, Fan J, McCandliss BD, et al Development of attentional networks in childhood. Neuropsychologia. 2004;42(8):1029–1040. 15093142 10.1016/j.neuropsychologia.2003.12.012

[73] Schmidgen J, Heinen T, Konrad K, Bender S. From preparation to post-processing: Insights into evoked and induced cortical activity during pre-cued motor reactions in children and adolescents. Neuroimage. 2024;297:120735. 39002787 10.1016/j.neuroimage.2024.120735

[74] Chevalier N, James TD, Wiebe SA, Nelson JM, Espy KA. Contribution of reactive and proactive control to children's working memory performance: Insight from item recall durations in response sequence planning. Dev Psychol. 2014;50(7):1999–2008. 24773104 10.1037/a0036644 PMC4222582

[75] Denckla MB . Development of speed in repetitive and successive finger-movements in normal children. Dev Med Child Neurol. 1973;15(5):635–645. 4765232 10.1111/j.1469-8749.1973.tb05174.x

[76] Largo RH, Fischer JE, Rousson V. Neuromotor development from kindergarten age to adolescence: Developmental course and variability. Swiss Med Wkly. 2003;133(13–14):193–199. 12811675 10.4414/smw.2003.09883

[77] Niechwiej-Szwedo E, Meier K, Christian L, et al Concurrent maturation of visuomotor skills and motion perception in typically-developing children and adolescents. Dev Psychobiol. 2020;62(3):353–367. 31621075 10.1002/dev.21931

[78] Adleman NE, Menon V, Blasey CM, et al A developmental fMRI study of the Stroop color-word task. Neuroimage. 2002;16(1):61–75. 11969318 10.1006/nimg.2001.1046

[79] Klingberg T . Development of a superior frontal-intraparietal network for visuo-spatial working memory. Neuropsychologia. 2006;44(11):2171–2177. 16405923 10.1016/j.neuropsychologia.2005.11.019

[80] Klingberg T, Forssberg H, Westerberg H. Increased brain activity in frontal and parietal cortex underlies the development of visuospatial working memory capacity during childhood. J Cogn Neurosci. 2002;14(1):1–10. 11798382 10.1162/089892902317205276

[81] Neufang S, Fink GR, Herpertz-Dahlmann B, Willmes K, Konrad K. Developmental changes in neural activation and psychophysiological interaction patterns of brain regions associated with interference control and time perception. Neuroimage. 2008;43(2):399–409. 18708149 10.1016/j.neuroimage.2008.07.039

[82] Gillebert CR, Op de Beeck HP, Panis S, Wagemans J. Subordinate categorization enhances the neural selectivity in human object-selective cortex for fine shape differences. J Cogn Neurosci. 2009;21(6):1054–1064. 18752400 10.1162/jocn.2009.21089

[83] Numssen O, Bzdok D, Hartwigsen G. Functional specialization within the inferior parietal lobes across cognitive domains. Elife. 2021;10:e63591. 33650486 10.7554/eLife.63591 PMC7946436

[84] Fogassi L, Luppino G. Motor functions of the parietal lobe. Curr Opin Neurobiol. 2005;15(6):626–631. 16271458 10.1016/j.conb.2005.10.015

[85] Grefkes C, Ritzl A, Zilles K, Fink GR. Human medial intraparietal cortex subserves visuomotor coordinate transformation. Neuroimage. 2004;23(4):1494–1506. 15589113 10.1016/j.neuroimage.2004.08.031

[86] Friedrich J, Verrel J, Kleimaker M, Munchau A, Beste C, Baumer T. Neurophysiological correlates of perception-action binding in the somatosensory system. Sci Rep. 2020;10(1):14794. 32908197 10.1038/s41598-020-71779-0 PMC7481208

[87] Borich MR, Brodie SM, Gray WA, Ionta S, Boyd LA. Understanding the role of the primary somatosensory cortex: Opportunities for rehabilitation. Neuropsychologia. 2015;79(Pt B)):246–255. 26164474 10.1016/j.neuropsychologia.2015.07.007 PMC4904790

[88] Vidoni ED, Acerra NE, Dao E, Meehan SK, Boyd LA. Role of the primary somatosensory cortex in motor learning: An rTMS study. Neurobiol Learn Mem. 2010;93(4):532–539. 20132902 10.1016/j.nlm.2010.01.011

[89] Kashiwagi M, Iwaki S, Narumi Y, Tamai H, Suzuki S. Parietal dysfunction in developmental coordination disorder: A functional MRI study. Neuroreport. 2009;20(15):1319–1324. 19730138 10.1097/WNR.0b013e32832f4d87

[90] Zapparoli L, Porta M, Gandola M, et al A functional magnetic resonance imaging investigation of motor control in Gilles de la Tourette syndrome during imagined and executed movements. Eur J Neurosci. 2016;43(4):494–508. 26566185 10.1111/ejn.13130

[91] Loibl M, Beutling W, Kaza E, Lotze M. Non-effective increase of fMRI-activation for motor performance in elder individuals. Behav Brain Res. 2011;223(2):280–286. 21569800 10.1016/j.bbr.2011.04.040

[92] Mattay VS, Fera F, Tessitore A, et al Neurophysiological correlates of age-related changes in human motor function. Neurology. 2002;58(4):630–635. 11865144 10.1212/wnl.58.4.630

[93] Riecker A, Groschel K, Ackermann H, Steinbrink C, Witte O, Kastrup A. Functional significance of age-related differences in motor activation patterns. Neuroimage. 2006;32(3):1345–1354. 16798017 10.1016/j.neuroimage.2006.05.021

[94] Elman JA, Oh H, Madison CM, et al Neural compensation in older people with brain amyloid-beta deposition. Nat Neurosci. 2014;17(10):1316–1318. 25217827 10.1038/nn.3806 PMC4177011

[95] Scheller E, Minkova L, Leitner M, Kloppel S. Attempted and successful compensation in preclinical and early manifest neurodegeneration—A review of task FMRI studies. Front Psychiatry. 2014;5:132. 25324786 10.3389/fpsyt.2014.00132 PMC4179340

[96] Wolf RC, Sambataro F, Vasic N, et al Abnormal resting-state connectivity of motor and cognitive networks in early manifest Huntington's disease. Psychol Med. 2014;44(15):3341–3356. 25066491 10.1017/S0033291714000579

[97] Gregory S, Long JD, Klöppel S, et al Testing a longitudinal compensation model in premanifest Huntington's disease. Brain. 2018;141(7):2156–2166. 29788038 10.1093/brain/awy122 PMC6022638

[98] Orth M, Munchau A, Rothwell JC. Corticospinal system excitability at rest is associated with tic severity in Tourette syndrome. Biol Psychiatry. 2008;64(3):248–251. 18243162 10.1016/j.biopsych.2007.12.009

[99] Ziemann U, Paulus W, Rothenberger A. Decreased motor inhibition in Tourette's disorder: Evidence from transcranial magnetic stimulation. Am J Psychiatry. 1997;154(9):1277–1284. 9286189 10.1176/ajp.154.9.1277

[100] Tajik-Parvinchi D, Sandor P. Enhanced antisaccade abilities in children with Tourette syndrome: The gap-effect reversal. Front Hum Neurosci. 2013;7:768. 24312038 10.3389/fnhum.2013.00768 PMC3826111

[101] Baumer T, Thomalla G, Kroeger J, et al Interhemispheric motor networks are abnormal in patients with Gilles de la Tourette syndrome. Mov Disord. 2010;25(16):2828–2837. 20960481 10.1002/mds.23418

[102] Bruce AB, Yuan W, Gilbert DL, et al Altered frontal-mediated inhibition and white matter connectivity in pediatric chronic tic disorders. Exp Brain Res. 2021;239(3):955–965. 33462641 10.1007/s00221-020-06017-0 PMC10340132

[103] Liao W, Yu Y, Miao HH, Feng YX, Ji GJ, Feng JH. Inter-hemispheric intrinsic connectivity as a neuromarker for the diagnosis of boys with Tourette syndrome. Mol Neurobiol. 2017;54(4):2781–2789. 27011382 10.1007/s12035-016-9863-9

[104] Beste C, Tubing J, Seeliger H, et al Altered perceptual binding in Gilles de la Tourette syndrome. Cortex. 2016;83:160–166. 27544346 10.1016/j.cortex.2016.07.015

[105] Kleimaker M, Takacs A, Conte G, et al Increased perception-action binding in Tourette syndrome. Brain. 2020;143(6):1934–1945. 32464659 10.1093/brain/awaa111

[106] Petruo V, Bodmer B, Brandt VC, et al Altered perception-action binding modulates inhibitory control in Gilles de la Tourette syndrome. J Child Psychol Psychiatry. 2019;60(9):953–962. 29924402 10.1111/jcpp.12938

[107] Chen X, Scangos KW, Stuphorn V. Supplementary motor area exerts proactive and reactive control of arm movements. J Neurosci. 2010;30(44):14657–14675. 21048123 10.1523/JNEUROSCI.2669-10.2010 PMC2990193

[108] Cunnington R, Bradshaw JL, Iansek R. The role of the supplementary motor area in the control of voluntary movement. Hum Mov Sci. 1996;15(5):627–647.

[109] Kasess CH, Windischberger C, Cunnington R, Lanzenberger R, Pezawas L, Moser E. The suppressive influence of SMA on M1 in motor imagery revealed by fMRI and dynamic causal modeling. Neuroimage. 2008;40(2):828–837. 18234512 10.1016/j.neuroimage.2007.11.040

[110] Cote SL, Elgbeili G, Quessy S, Dancause N. Modulatory effects of the supplementary motor area on primary motor cortex outputs. J Neurophysiol. 2020;123(1):407–419. 31774345 10.1152/jn.00391.2019 PMC6985856

[111] Nachev P, Kennard C, Husain M. Functional role of the supplementary and pre-supplementary motor areas. Nat Rev Neurosci. 2008;9(11):856–869. 18843271 10.1038/nrn2478

[112] Sumner P, Nachev P, Morris P, et al Human medial frontal cortex mediates unconscious inhibition of voluntary action. Neuron. 2007;54(5):697–711. 17553420 10.1016/j.neuron.2007.05.016 PMC1890004

[113] Tanji J, Hoshi E. Role of the lateral prefrontal cortex in executive behavioral control. Physiol Rev. 2008;88(1):37–57. 18195082 10.1152/physrev.00014.2007

[114] Fiske A, Holmboe K. Neural substrates of early executive function development. Dev Rev. 2019;52:42–62. 31417205 10.1016/j.dr.2019.100866 PMC6686207

[115] El Malhany N, Gulisano M, Rizzo R, Curatolo P. Tourette syndrome and comorbid ADHD: Causes and consequences. Eur J Pediatr. 2015;174(3):279–288. 25224657 10.1007/s00431-014-2417-0

