# Supplementary material for: Motor network organization in healthy development and chronic tic disorders
Source: Brain Commun. 2025 Jun 30;7(4):fcaf260. doi: 10.1093/braincomms/fcaf260 (PMC12264489; doi:10.1093/braincomms/fcaf260)
Supplement: fcaf260_Supplementary_Data [file fcaf260_supplementary_data.docx]

**Supplementary Material**

**Supplementary Table 1: Sample sizes per paradigm and analysis type**

| **Sample size** | **Healthy control subjects (HC)** | | **Patients and matched healthy controls (TD; HC)** | |
| --- | --- | --- | --- | --- |
|  | **Internal** | **External** | **Internal** | **External** |
| Behavioural | N = 52 | N = 55 | N = 21;17 | N = 21; 20 |
| fMRI | N = 45 | N = 43 | N = 14; 15 | N = 12; 15 |
| DCM | - | N = 24 | - | N = 10; 10 |

Columns on the left report sample sizes for the full healthy control sample. Columns on the right report sample sizes for patients and corresponding matched healthy control subsamples used in group comparisons. Sample sizes are shown separately for behavioural, fMRI, and DCM analyses to transparently reflect the final sample sizes at each analysis stage, after application of standard quality control procedures.

**Supplementary Table 2: Age distribution of healthy control subjects across analysis samples (External condition)**

| **Age group (Years)** | **Behavioural (N = 55)** | **fMRI (N = 43)** | **DCM (N = 24)** |
| --- | --- | --- | --- |
| 5-8 | N = 13 | N = 6 | N = 4 |
| 9-11 | N = 19 | N = 15 | N = 8 |
| 12-14 | N = 14 | N = 13 | N = 6 |
| 15-17 | N = 9 | N = 9 | N = 6 |

Age distributions are shown for the full healthy control sample, which was used for developmental analyses. Sample sizes are shown separately for behavioural, fMRI, and DCM analyses to transparently reflect the final sample sizes at each analysis stage, after application of standard quality control procedures.

**Statistical analysis of behavioural data**

Across all trials, RTs below 150 ms, exceeding 2000 ms and outliers beyond three standard deviations (SD) from individual averages per condition were excluded.^1^ Per condition, we determined participants’ average individual RTs to evaluate psychomotor speed. We calculated the percentage of correct responses (i.e., the proportion of correct responses relative to the total number of stimuli presented) to indicate task accuracy. For non-informative cues, error responses were defined as misses or false alarms. For the ‘External’ condition, misses, false alarms, and inaccurate responses (button presses on the side opposite to the one indicated by the arrow) were defined as error responses. When subjects pressed more than one button following the stimulus presentation, only the initial response was considered. To quantify blink reduction, we calculated the mean number of blinks across suppression and release blocks. Difference scores were derived by subtracting the mean number of blinks over the suppression blocks from the mean number of blinks over the release blocks. The absolute percentage of blink reduction across blocks was calculated by dividing the difference score by the mean number of blinks across the release blocks. To study the development of motor network functions, we explored the relationship between age (mean-centred) and task measures (RT, accuracy) using regression analyses, including linear, quadratic, and cubic models within the healthy sample. For clinical comparisons, group differences between TD patients and age-matched healthy controls were calculated using independent-samples t-test or non-parametric Mann-Whitney-U tests depending on data distribution.

**Supplementary Figure 1: Developmental trajectories of behavioural performance in the ‘Internal’ task-condition**


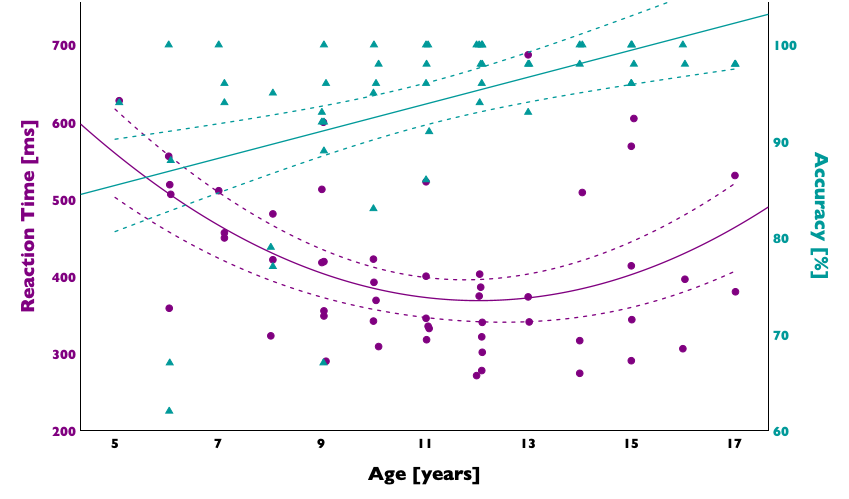


Developmental trajectory of internally cued responses in typically developing children and adolescents (N = 52). Regression analyses revealed linear age-related improvements in accuracy (teal; R^2^ = 0.240, B = 1.401, P < 0.001) and a steep decline in reaction times during early childhood (purple; R^2^ = 0.359, B = 3.475, P < 0.001). Reaction times are displayed in milliseconds (ms). Each data point represents one participant. Teal triangles represent individual accuracy values; purple circles represent individual reaction time values. Dashed lines indicate 95% confidence intervals for the fitted regression lines.

**fMRI data acquisition and analyses**

To minimize movement artifacts, participants were trained in a mock-scanner before the scanning session, receiving feedback on head-motion while practicing the fMRI paradigms in a realistic setting. Additionally, participants’ heads were fixated using foam pads surrounding the head. MRI scans were performed on a 3 Tesla Siemens MAGNETOM Prisma scanner (Siemens Healthcare, Erlangen, Germany). T1-weighted structural images were acquired by a magnetization-prepared rapid gradient echo (MP-RAGE) sequence (repetition time [T_R_] = 1790 ms, echo time [T_E_] = 2.53 ms, flip angle = 8°, number of slices = 176, slice thickness = 0.9 mm, interslice gap = 0.45 mm, field of view [FOV] = 256 mm, voxel size = 0.9 × 0.9 × 0.9 mm). Whole-brain T2-weighted functional images were obtained using an echoplanar imaging (EPI) multiband sequence, with blood oxygenation level-dependent (BOLD) contrast (T_R_ = 980 ms, T_E_ = 30 ms, flip angle = 70°, number of slices = 64, slice thickness = 2.0 mm, interslice gap = 0.2 mm, FOV = 207 mm, voxel size = 2.2 × 2.2 × 2.0 mm). Slices were acquired in transversal orientation in an interleaved order. Image pre-processing was performed using Statistical Parametric Mapping (SPM12; The Wellcome Centre for Human Neuroimaging, UCL Queen Square Institute of Neurology, London, UK [www.fil.ion.ucl.ac.uk/spm]) implemented in MATLAB (The MathWorks, Natick, USA). Pre-processing included slice timing about the middle slice, SPM12 standard realignment and unwarping to account for motion, co-registration to the mean EPI image, normalization to Montreal Neurological Institute (MNI) stereotactic space using unified segmentation based on the SPM tissue probability map for six tissue classes, and spatial smoothing with 8 mm full width at half-maximum isotropic Gaussian kernel, to reduce noise. Task-related blood oxygen level-dependent (BOLD) responses were modelled using the GLM framework, with contrasts capturing left- and right-handed movements (from the time of stimulus presentation until button presses) relative to baseline. Contrast images were produced as follows: ‘right-handed movements > resting baseline’ and ‘left-handed movements > resting baseline’. Model parameter estimates and t-statistic images were submitted to group-level analysis. Separate one-sample t-tests were conducted for healthy control subjects and TD patients to explore whole-brain task-related activations for these contrasts. For the healthy control group, effects were considered significant at a family-wise error (FWE) corrected voxel-level threshold of cluster-level PFWE < 0.05. For the TD patient group, effects were considered significant at an uncorrected voxel-level threshold of P < 0.001, with a cluster-level PFWE < 0.05. Furthermore, in healthy subjects, we examined the association between age and whole-brain activation from this contrast using regression analysis, adding mean-centred age, mean-centred age-squared and mean-centred age cubed as covariates. Additionally, we used independent-samples t-test to compare task-related activity from these contrasts between TD patients and healthy controls. For these analyses, effects were considered significant if they exceeded an uncorrected (P < 0.001) voxel-level threshold of cluster-level PFWE < 0.05. Cluster and local maxima labels were derived from the updated version of the AAL atlas 3 (AAL 3) ^2,3^ and further explored using the SPM Anatomy Toolbox, which assigns activations to the most likely cytoarchitectonic area by means of a maximum probability map.^4,5,6^

**Supplementary Table 3: Task activations healthy control subjects ‘Internal’: Left > Baseline**

**(P < 0.05 FWE)**

| **Local Maxima (Side)** | **x** | **y** | **z** | **Voxel** | **T-value** | **P_FWE-corr._** |
| --- | --- | --- | --- | --- | --- | --- |
| Fusiform gyrus (L) Cerebellum_6 (L) Cerebellum_Crus (L) | -36 | -68 | -18 | 11356 | 10.28 | < 0.001 |
| Postcentral gyrus (R) Precentral gyrus (R) Middle frontal gyrus (R) | 48 | -18 | 58 | 7158 | 9.76 | < 0.001 |
| Cuneus (R)  Superior occipital gyrus (R) Calcarine sulcus (R) | 16 | -104 | 10 | 517 | 8.87 | < 0.001 |
| Rolandic operculum (L) Insula (L) Frontal inferior operculum (L) | -44 | 0 | 10 | 792 | 8.70 | < 0.001 |
| Cerbellum_8 (R) Cerbellum_7b (R) Cerebellum_Crus1 | 30 | -58 | -48 | 218 | 6.28 | < 0.001 |
| Heschl’s gyrus (L) Insula (L) Rolandic operculum (L) | -36 | -20 | 8 | 24 | 6.03 | 0.007 |
| Precuneus (L) Mid cingulate cortex (L)  Paracentral lobule (L) | -8 | -46 | 60 | 20 | 5.87 | 0.008 |
| Precentral gyrus (R) Frontal inferior operculum (R) Inferior frontal gyrus (R) | 60 | 10 | 30 | 18 | 5.64 | 0.009 |
| Superior occipital gyrus (L) Cuneus (L) Calcarine sulcus (L) | -6 | -100 | 20 | 6 | 5.63 | 0.018 |
| Superior parietal gyrus (R) Postcentral gyrus (R) Precuneus (R) | 16 | -54 | 74 | 16 | 5.57 | 0.011 |
| Insula (R) Putamen (R) Heschl’s gyrus (R) | 36 | -18 | 6 | 22 | 5.48 | 0.007 |
| Precentral gyrus (L) Postcentral gyrus (L) Superior frontal gyrus (L) | -40 | -10 | 62 | 18 | 5.46 | 0.009 |
| Precuneus (R) Superior parietal gyrus (R) Precuneus (L) | 8 | -76 | 52 | 25 | 5.45 | 0.006 |
| Precuneus (L) Superior occipital gyrus (L) Superior parietal gyrus (L) | -12 | -66 | 36 | 16 | 5.44 | 0.011 |
| Calcarine sulcus (L) Lingual gyrus (L) Cerebellum_Crus1 (L) | -8 | -90 | -14 | 6 | 5.26 | 0.022 |

Per cluster, the table shows 3 local maxima > 8.0 mm apart

**Supplementary Table 4: Task activations healthy controls ‘Internal’: Right > Baseline**

**(P < 0.05 FWE)**

| **Local Maxima (Side)** | **x** | **y** | **z** | **Voxel** | **T-value** | **P_FWE-corr._** |
| --- | --- | --- | --- | --- | --- | --- |
| Precentral gyrus (L) Postcentral gyrus (L) Superior frontal gyrus (L) | -36 | -22 | 62 | 19106 | 12.08 | < 0.001 |
| Supplementary motor area (L) Mid cingulate gyrus (L) Supplementary motor area (R) | -6 | -6 | 56 | 1165 | 8.20 | < 0.001 |
| Supramarginal gyrus (R)  Superior temporal gyrus (R) Rolandic operculum (R) | 64 | -38 | 26 | 458 | 7.28 | < 0.001 |
| Insula (R) Frontal inferior operculum (R) Rolandic operculum (R) | 44 | 12 | -2 | 276 | 7.16 | < 0.001 |
| Middle temporal gyrus (R) Superior temporal gyrus (R) Hippocampus (R) | 48 | -26 | -8 | 15 | 5.73 | 0.009 |
| Middle occipital gyrus (L) Calcarine sulcus (L) Superior occipital gyrus (L) | -10 | -104 | 0 | 8 | 6.03 | 0.018 |
| Frontal inferior operculum (R) Inferior frontal gyrus (R)  Precentral gyrus (R) | 42 | 8 | 22 | 6 | 5.58 | 0.020 |
| Frontal inferior operculum (R) Rolandic operculum (R) Precentral gyrus (R) | 54 | 10 | 12 | 5 | 5.46 | 0.022 |
| Cerebellum_Crus2 (L) Cerebellum_7b (L) Cerebellum_8 (L) | -34 | -68 | -44 | 5 | 5.40 | 0.022 |

Per cluster, the table shows 3 local maxima > 8.0 mm apart

**Supplementary Table 5: Task activations healthy controls ‘External’: Left > Baseline (P < 0.05 FWE)**

| **Local Maxima (Side)** | **x** | **y** | **z** | **Voxel** | **T-value** | **P_FWE-corr._** |
| --- | --- | --- | --- | --- | --- | --- |
| Precentral gyrus (R) Postcentral gyrus (R) Middle frontal gyrus (R) | 48 | -18 | 62 | 34156 | 10.38 | < 0.001 |
| Insula (L) Rolandic operculum (L) Precentral gyrus (L) | -38 | -4 | 18 | 885 | 9.37 | < 0.001 |
| Cerebellum_8 (R)  Cerebellum_9 (R) Cerbellum_10 (R) | 28 | -50 | -46 | 73 | 7.11 | < 0.001 |
| Thalamus_PuM(R) Thalamus_PuL (R) Hippocampus (R) | 20 | -28 | 12 | 22 | 6.78 | 0.004 |
| Middle temporal gyrus (L) Superior temporal gyrus (L) Inferior temporal gyrus (L) | -64 | -48 | 12 | 84 | 6.32 | < 0.001 |
| Superior frontal gyrus (L) Middle frontal gyrus (L) Inferior frontal gyrus (L) | -30 | 48 | 40 | 19 | 6.13 | 0.005 |
| Mid cingulate gyrus (R) Posterior cingulate gyrus (R)  Posterior cingulate gyrus (L) | 8 | -38 | 32 | 9 | 6.04 | 0.012 |
| Precentral gyrus (L) Superior frontal gyrus (L) Middle frontal gyrus (L) | -38 | -6 | 64 | 36 | 6.03 | 0.001 |
| Paracentral lobule (R) Precuneus (R) Mid cingulate cortex (R) | 18 | -42 | 50 | 7 | 5.67 | 0.015 |
| Paracentral lobule (R) Precuneus (R) Postcentral gyrus (R) | 8 | -38 | 58 | 14 | 5.65 | 0.008 |

Per cluster, the table shows 3 local maxima > 8.0 mm apart

**Supplementary Table 6: Task activations healthy controls ‘External’: Right > Baseline**

**(P < 0.05 FWE)**

| **Local Maxima (Side)** | **x** | **y** | **z** | **Voxel** | **T-value** | **P_FWE-corr._** |
| --- | --- | --- | --- | --- | --- | --- |
| Precentral gyrus (L) Postcentral gyrus (L) Superior parietal gyrus (L) | -38 | -30 | 70 | 32183 | 12.30 | < 0.001 |
| Inferior frontal gyrus (R) Precentral gyrus (R) Inferior frontal gyrus (R) | 64 | 12 | 22 | 1419 | 8.87 | < 0.001 |
| Cerebellum_8 (L)  Cerebellum_7b (L) Cerbellum_Crus2 (L) | -36 | -58 | -48 | 83 | 7.03 | < 0.001 |
| Superior parietal gyrus (L) Precuneus (R) Postcentral gyrus (R) | 14 | -52 | 62 | 263 | 6.98 | 0.004 |
| Middle frontal gyrus (R) Superior frontal gyrus (R) Inferior frontal gyrus (R) | 36 | 50 | 36 | 189 | 6.74 | < 0.001 |
| Cerebellum_Crus2 (L) Cerebellum_7b (L) Cerebellum_8 (L) | -14 | -76 | -40 | 43 | 6.35 | 0.001 |
| Middle frontal gyrus (L) Superior frontal gyrus (L)  Inferior frontal gyrus (L) | -32 | 48 | 38 | 48 | 6.02 | 0.001 |
| Supplementary motor area (R) Superior frontal gyrus (R) Supplementary motor area (L) | 12 | 6 | 74 | 6 | 5.85 | 0.016 |
| Precuneus (L) Superior parietal gyrus (L) Precuneus (R) | -6 | -66 | 48 | 33 | 5.85 | 0.001 |
| Insula (L) Inferior frontal gyrus (L) Middle frontal gyrus (L) | -28 | 32 | 10 | 5 | 5.69 | 0.019 |
| Supramarginal gyrus (R) Inferior parietal gyrus (R) Postcentral gyrus (R) | 32 | -42 | 44 | 9 | 5.67 | 0.012 |

Per cluster, the table shows 3 local maxima > 8.0 mm apart

**Supplementary Table 7: Task activations patients ‘Internal’: Left > Baseline (P < 0.001 unc.)**

| **Local Maxima (Side)** | **x** | **y** | **z** | **Voxel** | **T-value** | **P_FWE-corr._** |
| --- | --- | --- | --- | --- | --- | --- |
| Precentral gyrus (R) Postcentral gyrus (R) Superior frontal gyrus (L) | 34 | -20 | 58 | 664 | 5.85 | 0.004 |

Per cluster, the table shows 3 local maxima > 8.0 mm apart

**Supplementary Table 8: Task activations patients ‘Internal’: Right > Baseline (P < 0.001 unc.)**

| **Local Maxima (Side)** | **x** | **y** | **z** | **Voxel** | **T-value** | **P_FWE-corr._** |
| --- | --- | --- | --- | --- | --- | --- |
| Inferior occipital gyrus (R) Middle occipital gyrus (R) Inferior temporal gyrus (R) | 46 | -84 | -8 | 5989 | 14.78 | < 0.001 |
| Precentral gyrus (L) Superior frontal gyrus (L) Postcentral gyrus (L) | -40 | -12 | 70 | 8915 | 12.13 | < 0.001 |
| Middle frontal gyrus (L)  Superior frontal gyrus (L) Inferior frontal gyrus (L) | -36 | 50 | 20 | 932 | 8.34 | < 0.001 |
| Cuneus (R) Superior occipital gyrus (R) Cuneus (L) | 12 | -94 | 24 | 401 | 7.48 | 0.005 |
| Postcentral gyrus (R) Supramarginal gyrus (R) Precentral gyrus (R) | 64 | -18 | 50 | 946 | 7.21 | < 0.001 |
| Postcentral gyrus (R) Superior parietal gyrus (R) Inferior parietal gyrus (R) | 34 | -42 | 62 | 328 | 6.20 | 0.012 |
| Supramarginal gyrus (R) Postcentral gyrus (R)  Rolandic operculum (R) | 46 | -22 | 28 | 350 | 5.35 | 0.009 |

Per cluster, the table shows 3 local maxima > 8.0 mm apart

**Supplementary Table 9: Task activations patients ‘External’: Left > Baseline (P < 0.001 unc.)**

| **Local Maxima (Side)** | **x** | **y** | **z** | **Voxel** | **T-value** | **P_FWE-corr._** |
| --- | --- | --- | --- | --- | --- | --- |
| Vermis_6 Vermis_4_5 Vermis_8 | -2 | -58 | -24 | 226 | 7.04 | 0.036 |
| Cerebellum_9 (L) Cerebellum_8 (L) Vermis_9 | -10 | -60 | -44 | 227 | 6.71 | 0.035 |

Per cluster, the table shows 3 local maxima > 8.0 mm apart.

**Supplementary Table 10: Task activations patients ‘External’: Right > Baseline (P < 0.001 unc.)**

| **Local Maxima (Side)** | **x** | **y** | **z** | **Voxel** | **T-value** | **P_FWE-corr._** |
| --- | --- | --- | --- | --- | --- | --- |
| Precuneus (L) Precuneus (R) Superior parietal gyrus (L) | -6 | -62 | 58 | 27637 | 11.40 | < 0.001 |
| Postcentral gyrus (R) Supramarginal gyrus (R) Precentral gyrus (R) | 42 | -28 | 38 | 1843 | 9.21 | < 0.001 |
| Medial orbitofrontal cortex (R) Medial orbitofrontal cortex (L)  Rectus (R) | 4 | 60 | -12 | 183 | 7.21 | 0.041 |
| Anterior cingulate cortex (L) Anterior cingulate cortex (L) Superior frontal gyrus (L) | -8 | 38 | 18 | 244 | 6.80 | 0.012 |
| Rolandic operculum (R) Heschl’s gyrus (R) Superior temporal gyrus (R) | 68 | 0 | 8 | 298 | 6.71 | 0.005 |

Per cluster, the table shows 3 local maxima > 8.0 mm apart.

**Dynamic Causal Modelling (DCM)**

DCM is a Bayesian framework incorporated into SPM12, designed to deduce hidden neuronal states based on measurements of brain activation. It can be applied to identify connectivity strengths among neuronal groups, to investigate how these connections change over time and how they are modulated depending on the context.^7^

DCM was applied to explore interhemispheric motor-network connectivity, following Michely et al.,^8^ who used the paradigm to examine age-related connectivity changes in healthy adults. The same regions of interest were used to assess neural interactions across developmental stages. We specified nine ROIs for the interhemispheric DCM model: 1. left PFC, 2. right PFC, 3. left PMC, 4. right PMC, 5. SMA, 6. left M1, 7. right M1, 8. left IPS, 9. right IPS (see Fig. 2). Time series were extracted from subject-specific coordinates defined in the ‘External’ condition. Within an 8-mm-radius sphere around the group peak coordinates, which were set as origin (see Table 10), we located the nearest individual activation peak coordinates from each subject’s first level GLM-analysis (see Table 11 and Table 12 for group mean coordinates). We extracted the first eigenvariate of the individual BOLD time series. For extraction of time series, we employed a threshold of P < 0.05 (uncorrected). Following recommendations by Zeidman et al.,^9^ for handling cases where ROIs showed no significant voxel response at this threshold, a stepwise lowering of the threshold was conducted in steps of 0.05, until a peak was discernible.

**Supplementary Figure 2: Model space for the Bayesian model selection procedure**


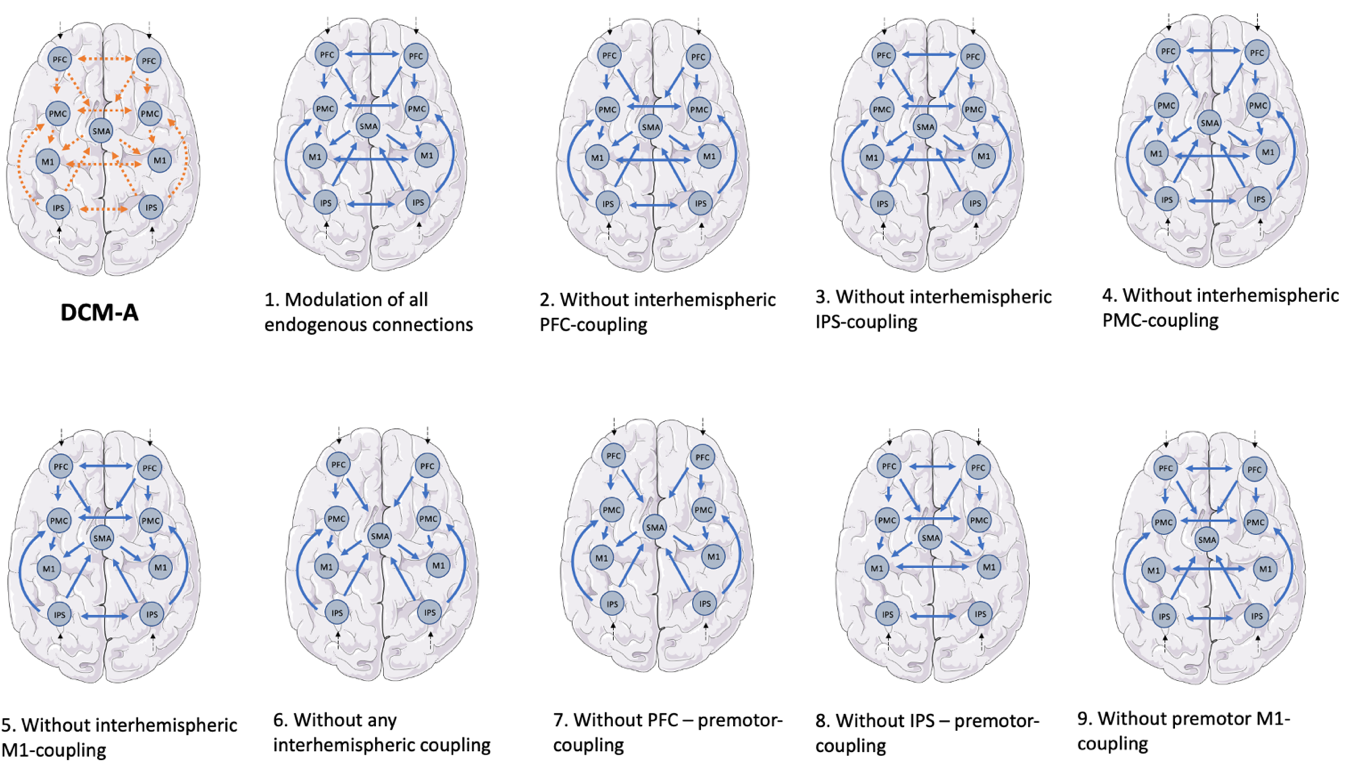


Each panel illustrates a distinct model tested using Bayesian model selection (BMS). The endogenous connectivity matrix is displayed in orange (top-left, DCM-A), while task-based connectivity matrices (DCM-B) are shown in blue across Models 1-9. Each model is labelled according to the connections excluded (e.g., Model 2: without interhemispheric PFC-PFC coupling). Arrows indicate directional connections between predefined regions of interest. PFC = prefrontal cortex; IPS = intraparietal sulcus; PMC = premotor cortex; M1 = primary motor cortex; SMA = supplementary motor area.

**Supplementary Figure 3: Model evidence for the selection of winning models**

**Healthy controls TD patients + matched controls**


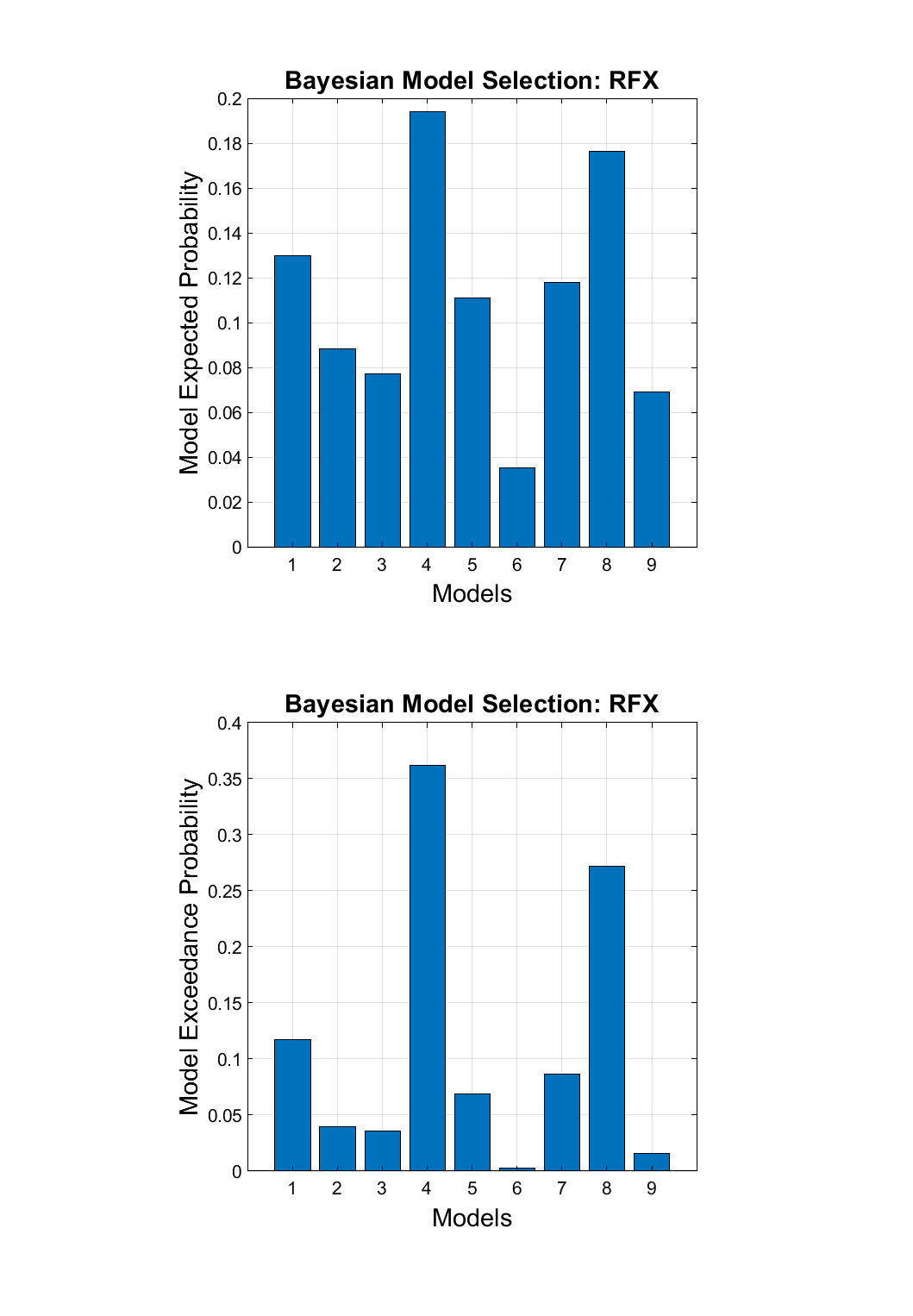

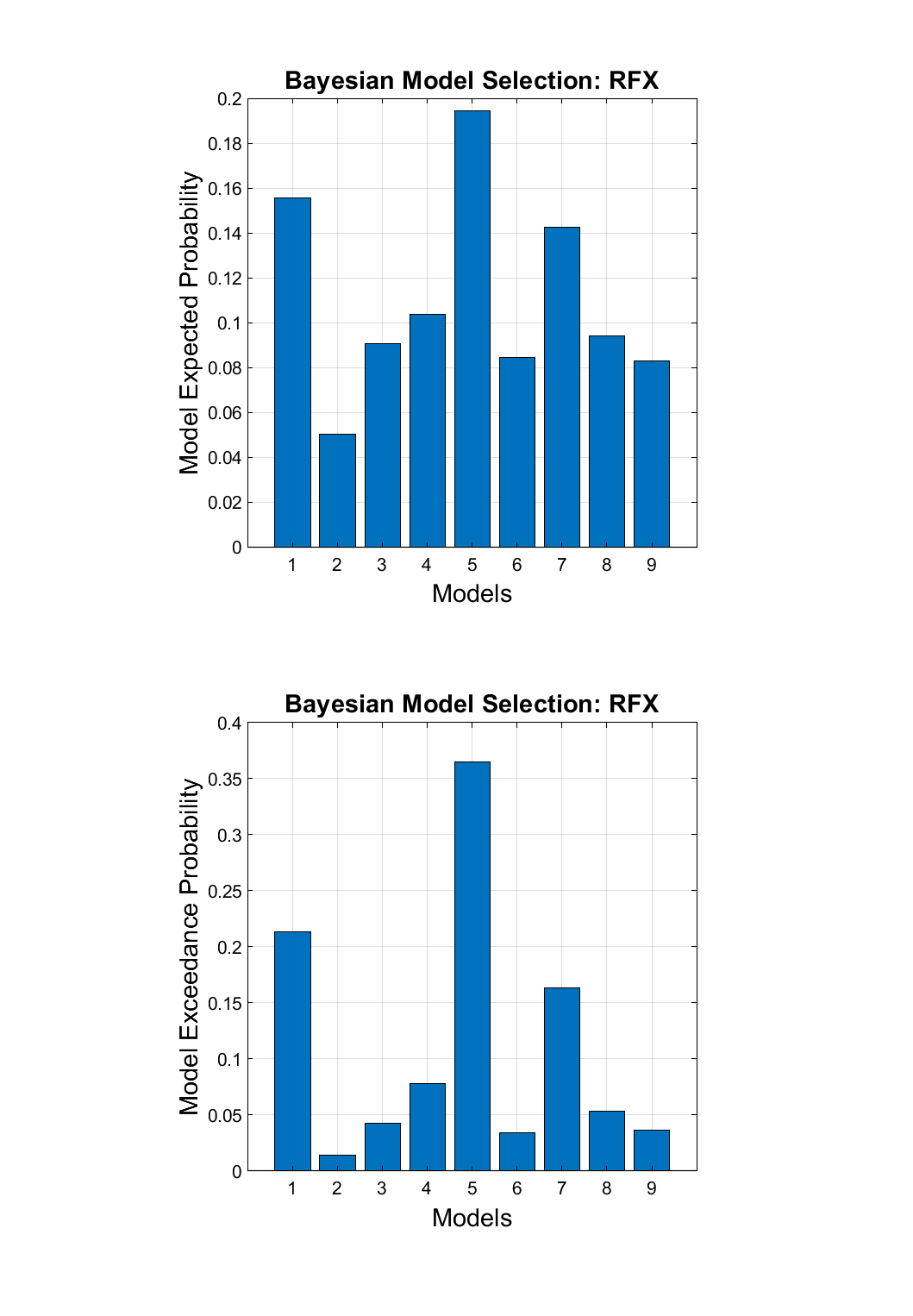


**Left:** Winning model for the group of healthy control subjects (N = 24) identified via Bayesian model selection (Model 4; without interhemispheric PMC coupling). **Right:** Winning model for TD patients and matched control subjects (N = 20; Model 5; without interhemispheric M1 coupling). Exceedance probabilities are shown as bar graphs. RFX = random-effects analysis; PMC = premotor cortex; M1 = primary motor cortex.

**Supplementary Table 11: Group peak coordinates used as origin for ROI extraction**

| **Region** | **x-coordinate** | **y-coordinate** | **z-coordinate** |
| --- | --- | --- | --- |
| PFC (L) | -32 | 48 | 38 |
| PFC (R) | 36 | 54 | 32 |
| SMA | -4 | -4 | 72 |
| PMC (L) | -32 | -18 | 72 |
| PMC (R) | 12 | 4 | 74 |
| M1 (L) | -36 | -28 | 60 |
| M1 (R) | 38 | -24 | 72 |
| IPS (L) | -36 | -46 | 54 |
| IPS (R) | 54 | -32 | 44 |

L = left; R = right; PFC = prefrontal cortex; SMA = supplementary motor area; PMC = premotor cortex; M1 = primary motor cortex; IPS = intraparietal sulcus.

**Supplementary Table 12: ROI coordinates (group mean)**

| **Region** | **Healthy control subjects** | | | **Patients and matched controls** | | | |
| --- | --- | --- | --- | --- | --- | --- | --- |
|  | **x- coordinate** | **y-coordinate** | **z-coordinate** | **x-coordinate** | | **y-coordinate** | **z-coordinate** |
| PFC (L) | -31.9 (± 3.1) | 47.5 (± 3.0) | 38.0 (± 3.3) | -32.6 (± 2.6) | 47.7 (± 2.8) | | 38.3 (± 3.2) |
| PFC (R) | 35.0 (± 2.0) | 53.5 (± 2.8) | 32.2 (± 2.3) | 36.4 (± 2.3) | 53.6 (± 3.1) | | 31.7 (± 2.1) |
| SMA | -3.7 (± 1.8) | -4.3 (± 2.2) | 58.2 (± 2.5) | -4.2 (± 1.8) | -4.1 (± 1.3) | | 58.9 (± 2.6) |
| PMC (L) | -32.1 (± 1.6) | -18.3 (± 0.7) | 71.6 (± 1.4) | -32.2 (± 1.4) | -18.0 (± 1.3) | | 71.6 (± 1.3) |
| PMC (R) | 11.3 (± 3.2) | 3.6 (± 2.8) | 73.0 (± 3.4) | 11.4 (± 3.0) | 3.8 (± 2.8) | | 72.5 (± 2.8) |
| M1 (L) | -36.4 (± 1.2) | -27.9 (± 1.9) | 60.8 (± 1.8) | -36.5 (± 1.4) | -28.2 (± 1.7) | | 60.2 (± 1.6) |
| M1 (R) | 32.5 (± 3.1) | -23.8 (± 3.9) | 70.9 (± 2.3) | 37.7 (± 2.4) | -23.5 (± 3.5) | | 70.9 (± 2.4) |
| IPS (L) | -35.6 (± 2.0) | -45.2 (± 3.6) | 54.8 (± 2.5) | -35.4 (± 2.4) | -45.8 (± 3.0) | | 54.9 (± 2.5) |
| IPS (R) | 54.2 (± 2.4) | -31.7 (± 3.4) | 43.7 (± 3.0) | 54.1 (± 2.8) | -30.8 (± 2.4) | | 43.5 (± 2.3) |

Standard deviation in parentheses; L = left; R = right; PFC = prefrontal cortex; SMA = supplementary motor area; PMC = premotor cortex; M1 = primary motor cortex; IPS = intraparietal sulcus.

**Supplementary Table 13: Coupling strengths of healthy control subjects (group mean)**

| **Coupling Parameters** | **Endogenous connections (DCM-A)** | | **Task-based connections (DCM-B)** | |
| --- | --- | --- | --- | --- |
|  | **Mean** | **SEM** | **Mean** | **SEM** |
| PFC (L) – PFC (R) | 0.199 | 0.041 | 0.143 | 0.249 |
| PFC (L) – PMC (L) | - 0.438 | 0.019 | 0.428 | 1.282 |
| PFC (L) – SMA | - 0.006 | 0.020 | 0.165 | 0.104 |
| PFC (R) – PFC (L) | 0.072 | 0.020 | 0.315 | 0.204 |
| PFC (R) – PMC (R) | 0.095 | 0.037 | 0.315 | 0.204 |
| PFC (R) – SMA | 0.033 | 0.021 | 0.083 | 0.095 |
| PMC (L) – PMC (R) | 0.079 | 0.034 |  |  |
| PMC (L) – M1(L) | 0.022 | 0.022 | 0.502 | 0.083 |
| PMC (R) – PMC (L) | 0.125 | 0.041 |  |  |
| PMC (R) – M1 (R) | 0.061 | 0.026 | 0.132 | 0.253 |
| SMA – M1 (L) | 0.038 | 0.021 | 0.308 | 0.209 |
| SMA – M1 (R) | 0.112 | 0.045 | 0.206 | 0.287 |
| M1 (L) – M1 (R) | 0.273 | 0.029 | 0.356 | 0.215 |
| M1 (R) – M1 (L) | 0.106 | 0.035 | 0.816 | 0.240 |
| IPS (L) – PMC (L) | - 0.078 | 0.044 | 0.425 | 0.374 |
| IPS (L) - SMA | 0.009 | 0.025 | 0.353 | 0.187 |
| IPS (L) IPS (R) | - 0.017 | 0.055 | - 0.334 | 0.347 |
| IPS (R) – PMC (R) | 0.038 | 0.046 | 0.070 | 0.239 |
| IPS (R) – SMA | 0.061 | 0.035 | 0.282 | 0.159 |
| IPS (R) – IPS (L) | - 0.057 | 0.075 | - 0.051 | 0.350 |

SEM = Standard error of the mean L = left; R = right; PFC = prefrontal cortex; SMA = supplementary motor area; PMC = premotor cortex; M1 = primary motor cortex; IPS = intraparietal sulcus.

**Supplementary Table 14: Coupling strengths of patients and matched control subjects
(group mean endogenous connections)**

| **Coupling Parameters** | **TD Patients** | | **Matched control subjects** | |
| --- | --- | --- | --- | --- |
|  | **Mean** | **SEM** | **Mean** | **SEM** |
| PFC (L) – PFC (R) | 0.015 | 0.052 | 0.002 | 0.043 |
| PFC (L) – PMC (L) | - 0.015 | 0.041 | - 0.049 | 0.033 |
| PFC (L) – SMA | 0.001 | 0.015 | - 0.002 | 0.033 |
| PFC (R) – PFC (L) | 0.050 | 0.057 | 0.020 | 0.107 |
| PFC (R) – PMC (R) | 0.008 | 0.039 | - 0.002 | 0.055 |
| PFC (R) – SMA | 0.001 | 0.021 | - 0.043 | 0.067 |
| PMC (L) – PMC (R) | 0.032 | 0.045 | 0.019 | 0.076 |
| PMC (L) – M1(L) | 0.070 | 0.067 | 0.001 | 0.045 |
| PMC (R) – PMC (L) | 0.089 | 0.085 | - 0.006 | 0.035 |
| PMC (R) – M1 (R) | 0.025 | 0.068 | 0.102 | 0.056 |
| SMA – M1 (L) | 0.053 | 0.056 | 0.051 | 0.052 |
| SMA – M1 (R) | 0.179 | 0.083 | 0.122 | 0.047 |
| M1 (L) – M1 (R) | 0.070 | 0.034 | 0.042 | 0.128 |
| M1 (R) – M1 (L) | 0.170 | 0.089 | 0.060 | 0.025 |
| IPS (L) – PMC (L) | - 0.106 | 0.042 | 0.053 | 0.056 |
| IPS (L) - SMA | - 0.040 | 0.063 | 0.070 | 0.066 |
| IPS (L) IPS (R) | - 0.098 | 0.146 | 0.160 | 0.096 |
| IPS (R) – PMC (R) | 0.041 | 0.072 | - 0.024 | 0.662 |
| IPS (R) – SMA | 0.034 | 0.058 | - 0.071 | 0.567 |
| IPS (R) – IPS (L) | - 0.114 | 0.085 | 0.084 | 0.084 |

SEM = Standard error of the mean; L = left; R = right; PFC = prefrontal cortex; SMA = supplementary motor area; PMC = premotor cortex; M1 = primary motor cortex; IPS = intraparietal sulcus.

**Supplementary Table 15: Coupling strengths of patients and matched control subjects
(group mean task-based connections)**

| **Coupling Parameters** | **TD Patients** | | **Matched control subjects** | |
| --- | --- | --- | --- | --- |
|  | **Mean** | **SEM** | **Mean** | **SEM** |
| PFC (L) – PFC (R) | - 0.239 | 0.428 | 0.585 | 0.585 |
| PFC (L) – PMC (L) | 0.022 | 0.119 | 0.199 | 0.235 |
| PFC (L) – SMA | 0.066 | 0.138 | 0.570 | 0.285 |
| PFC (R) – PFC (L) | 0.320 | 0.172 | 0.330 | 0.282 |
| PFC (R) – PMC (R) | 0.297 | 0.238 | 0.167 | 0.231 |
| PFC (R) – SMA | 0.433 | 0.320 | 0.290 | 0.173 |
| PMC (L) – PMC (R) | - 0.195 | 0.401 | 0.925 | 0.594 |
| PMC (L) – M1(L) | 0.574 | 0.309 | 0.990 | 0.512 |
| PMC (R) – PMC (L) | - 0.186 | 0.415 | - 0.047 | 0.988 |
| PMC (R) – M1 (R) | 0.529 | 0.271 | 0.124 | 0.268 |
| SMA – M1 (L) | 0.143 | 0.335 | 0.517 | 0.162 |
| SMA – M1 (R) | 0.154 | 0.361 | 0.128 | 0.312 |
| M1 (L) – M1 (R) |  |  |  |  |
| M1 (R) – M1 (L) |  |  |  |  |
| IPS (L) – PMC (L) | 0.218 | 0.320 | 0.419 | 0.450 |
| IPS (L) - SMA | 0.160 | 0.238 | 0.209 | 0.184 |
| IPS (L) IPS (R) | - 0.537 | 0.647 | 1.538 | 0.763 |
| IPS (R) – PMC (R) | 0.127 | 0.337 | 0.019 | 0.513 |
| IPS (R) – SMA | 0.448 | 0.375 | 0.777 | 0.424 |
| IPS (R) – IPS (L) | - 0.363 | 0.404 | 0.450 | 0.672 |

SEM = Standard error of the mean; L = left; R = right; PFC = prefrontal cortex; SMA = supplementary motor area; PMC = premotor cortex; M1 = primary motor cortex; IPS = intraparietal sulcus.

**References**

1. Michely J, Volz LJ, Barbe MT, et al. Dopaminergic modulation of motor network dynamics in Parkinson's disease. *Brain*. Mar 2015;138(Pt 3): 664-678. <https://doi.org/10.1093/brain/awu381>

2. Rolls ET, Huang, C-C, Lin C.P, Feng J, Joliot, M. Automated anatomical labelling atlas 3. *Neuroimage*. 2020/02/01/2020;206:116189. <https://doi.org/https://doi.org/10.1016/j.neuroimage.2019.116189>

3. Tzourio-Mazoyer N, Landeau B, Papathanassiou D, et al. Automated Anatomical Labeling of Activations in SPM Using a Macroscopic Anatomical Parcellation of the MNI MRI Single Subject Brain. *Neuroimage*. 2002/01/01/2002;15(1), 273-289. <https://doi.org/https://doi.org/10.1006/nimg.2001.0978>

4. Eickhoff SB, Heim S, Zilles K, Amunts K. Testing anatomically specified hypotheses in functional imaging using cytoarchitectonic maps. *Neuroimage*. Aug 15 2006; 32(2):570-582. <https://doi.org/10.1016/j.neuroimage.2006.04.204>

5. Eickhoff SB, Paus T, Caspers S, et al. Assignment of functional activations to probabilistic cytoarchitectonic areas revisited. *Neuroimage*. Jul 1 2007;36(3):511-521. <https://doi.org/10.1016/j.neuroimage.2007.03.060>

6. Eickhoff SB, Stephan KE, Mohlberg H, et al. A new SPM toolbox for combining probabilistic cytoarchitectonic maps and functional imaging data. *Neuroimage*. May 2005;25(4):1325-1335. <https://doi.org/10.1016/j.neuroimage.2004.12.034>

7. Stephan KE, Penny WD, Daunizeau J, Moran RJ, Friston KJ. Bayesian model selection for group studies. *Neuroimage*. Jul 15 2009;46(4):1004-1017. <https://doi.org/10.1016/j.neuroimage.2009.03.025>

8. Michely J, Volz LJ, Hoffstaedter F, et al. Network connectivity of motor control in the ageing brain. *Neuroimage Clin*. 2018;18, 443-455. <https://doi.org/10.1016/j.nicl.2018.02.001>

9. Zeidman P, Jafarian A, Corbin, N, et al. A guide to group effective connectivity analysis, part 1: First level analysis with DCM for fMRI. *Neuroimage*. Oct 15 2019;200, 174-190. <https://doi.org/10.1016/j.neuroimage.2019.06.031>
